# The effect of engaging unpaid informal providers on case detection and treatment initiation rates for TB and HIV in rural Malawi (Triage Plus): A cluster randomised health system intervention trial

**DOI:** 10.1371/journal.pone.0183312

**Published:** 2017-09-06

**Authors:** George Bello, Brian Faragher, Lifah Sanudi, Ireen Namakhoma, Hastings Banda, Rasmus Malmborg, Rachael Thomson, S. Bertel Squire

**Affiliations:** 1 Research for Equity And Community Health (REACH) Trust, Lilongwe, Malawi; 2 Centre for Applied Health Research & Delivery, Liverpool School of Tropical Medicine, Pembroke Place, Liverpool, United Kingdom; 3 LHL International Tuberculosis Foundation, Oslo, Norway; TNO, NETHERLANDS

## Abstract

**Background:**

The poor face barriers in accessing services for tuberculosis (TB) and Human Immuno-deficiency Virus (HIV) disease. A cluster randomised trial was conducted to investigate the effectiveness of engaging unpaid informal providers (IPs) to promote access in a rural district. The intervention consisted of training unpaid IPs in TB and HIV disease recognition, sputum specimen collection, appropriate referrals, and raising community awareness.

**Methods:**

In total, six clusters were defined in the study areas. Through a pair-matched cluster randomization process, three clusters (average cluster population = 200,714) were allocated to receive the intervention in the Early arm. Eleven months later the intervention was rolled out to the remaining three clusters (average cluster population = 209,564)—the Delayed arm. Treatment initiation rates for TB and Anti-Retroviral Therapy (ART) were the primary outcome measures. Secondary outcome measures included testing rates for TB and HIV. We report the results of the comparisons between the Early and Delayed arms over the 23 month trial period. Data were obtained from patient registers. Poisson regression models with robust standard errors were used to express the effectiveness of the intervention as incidence rate ratios (IRR).

**Results:**

The Early and Delayed clusters were well matched in terms of baseline monthly mean counts and incidence rate ratios for TB and ART treatment initiation. However there were fewer testing and treatment initiation facilities in the Early clusters (TB treatment n = 2, TB testing n = 7, ART initiation n = 3, HIV testing n = 20) than in the Delayed clusters (TB treatment n = 4, TB testing n = 9, ART initiation n = 6, HIV testing n = 18). Overall there were more HIV testing and treatment centres than TB testing and treatment centres. The IRR was 1.18 (95% CI: 0.903–1.533; p = 0.112) for TB treatment initiation and 1.347 (CI:1.00–1.694; p = 0.049) for ART initiation in the first 12 months and the IRR were 0.552 (95% CI:0.397–0.767; p<0.001) and 0.924 (95% CI: 0.369–2.309, p = 0.863) for TB and ART treatment initiations respectively for the last 11 months. The IRR were 1.152 (95% CI:1.009–1.359, p = 0.003) and 1.61 (95% CI:1.385–1.869, p<0.001) for TB and HIV testing uptake respectively in the first 12 months. The IRR was 0.659 (95% CI:0.441–0.983; p = 0.023) for TB testing uptake for the last 11 months.

**Conclusions:**

We conclude that engagement of unpaid IPs increased TB and HIV testing rates and also increased ART initiation. However, for these providers to be effective in promoting TB treatment initiation, numbers of sites offering TB testing and treatment initiation in rural areas should be increased.

**Trial registration:**

ClinicalTrials.gov NCT02127983.

## Introduction

Despite global efforts to improve tuberculosis (TB) case detection and reduce TB incidence, TB case detection rates remain below the global target of at least 70% [1]. Access to timely TB services is complicated by both system and patient barriers such as delays and repeated visits to multiple care providers, which in turn increases patient drop-out in the course of care seeking[2–5]. A study conducted in Malawi to measure the extent of, and factors associated with, patient delay in seeking health care, as well as health system delay in the diagnosis and treatment of new and retreatment TB cases, showed that occupation status and lack of knowledge of the fact that more than three weeks of coughing is a symptom of TB, were significantly associated with patient delay[5]. Furthermore, health system barriers, such as incorrect management of patients with negative smears and health workers failing to correctly identify all TB patients in health care settings, contribute to delays in timely TB case detection and treatment initiations[6,7].

Delay in correct diagnosis and treatment of TB leads to increased mortality due to increased disease severity and complications [8]. Active case finding has been used to improve TB case detection rates though its effectiveness still remains uncertain [9]. However, studies conducted in Harare, Zimbabwe, demonstrated that the use of community based active case finding approaches in high TB and HIV prevalence settings improved TB case detection rates [10]. Nevertheless, the cost of active case finding approaches hinders sustainability of such programmes especially in resource-limited settings [11]. Innovative approaches for improving TB case detection in the general population are thus required. Given that community management of undiagnosed tuberculosis remains a challenge [12,13], and that most new tuberculosis infections occur outside households in high incidence settings [14], adopting interventions that involve community participation is necessary for reducing tuberculosis transmission [15].

With TB in Sub-Saharan Africa being worsened because of its interaction with HIV and AIDS(1), integrated interventions for TB and HIV are advocated as approaches to combat the dual diseases especially in clinic based management [16]. Of the 9.27 million TB cases in 2007 globally, 15% (1.37 million) cases were HIV-related tuberculosis, of which 79% were from the African region(1). In 2012, an estimated 1.1 million (13%) of the 8.6 million TB cases were HIV positive and about 75% of the TB cases were from the African Region [17].

According to the WHO interim policy on collaborative TB and HIV activities [18], TB and HIV clinic based interventions can be integrated in two ways. First, tuberculosis services may be integrated into HIV health-care settings in order to reduce the TB burden among people living with HIV. Second, HIV services may be integrated into tuberculosis control programmes with a view to reducing the HIV burden in patients with TB.

Studies conducted to assess the effectiveness of the clinic based integration of TB and HIV in service provision for co-infected individuals have shown TB and HIV service integration to be effective in improving TB and HIV health outcomes and reducing the delay in treatment initiation [19,20]. However, given the structural barriers faced by the poor in accessing TB and HIV services in health facilities, the use of clinic based integration of TB and HIV services is necessary, but not sufficient, to sustainably address the TB and HIV epidemics, and therefore needs to be complemented with community based strategies involving informal health care providers such as storekeepers, traditional healers or village health committees.

Informal healthcare providers are known to be the first point of call along the health-seeking pathway for the most poor and vulnerable and play a critical role in reaching individuals who might otherwise not access health services because they are less served by the formal health system. The use of informal healthcare providers in community interventions to improve access to and coverage of basic health services is well documented [21–23]. For instance, in Ethiopia extension health workers have been used in TB identification and sputum collection for TB diagnosis which resulted in improved case detection, 122.2% in intervention areas compared to 69.4% in control areas [21]. Previous work in this area has mainly focused on engagement of paid, close-to-community health care workers which may not be a sustainable approach in resource limited settings. To address this research gap, a cluster randomised intervention trial involving non-paid informal health care providers called ‘Triage Plus’ was implemented in rural Malawi. The overall aim of our trial was, therefore, to investigate the effectiveness of engaging informal healthcare providers in an integrated TB and HIV community intervention on testing and treatment initiation rates.

## Methods

### Ethical approvals and standards of care

Research approval was granted by the Malawi Health Sciences Research and Ethics Committee, and the Research Ethics Committee of the Liverpool School of Tropical Medicine, UK on 8^th^ July 2008 and 10^th^ September 2008 respectively. Because data were collected from routine patient registers, no individualised consent was obtained from the patient participants. Consent to collect the data from registers was obtained from the national level (National TB Programme, and HIV Department), district level authorities and the respective managers of the health facilities. Patients visiting health facilities for TB and HIV services were managed by Ministry of Health workers according to existing standard guidelines. Training of informal providers and collection of data from registers were initiated after obtaining ethical approvals from the two ethical boards but before the registration of the trial due to an oversight on the part of the study team. The authors confirm that all ongoing and related trials for this intervention are registered.

### Study population

**Fig 1:**
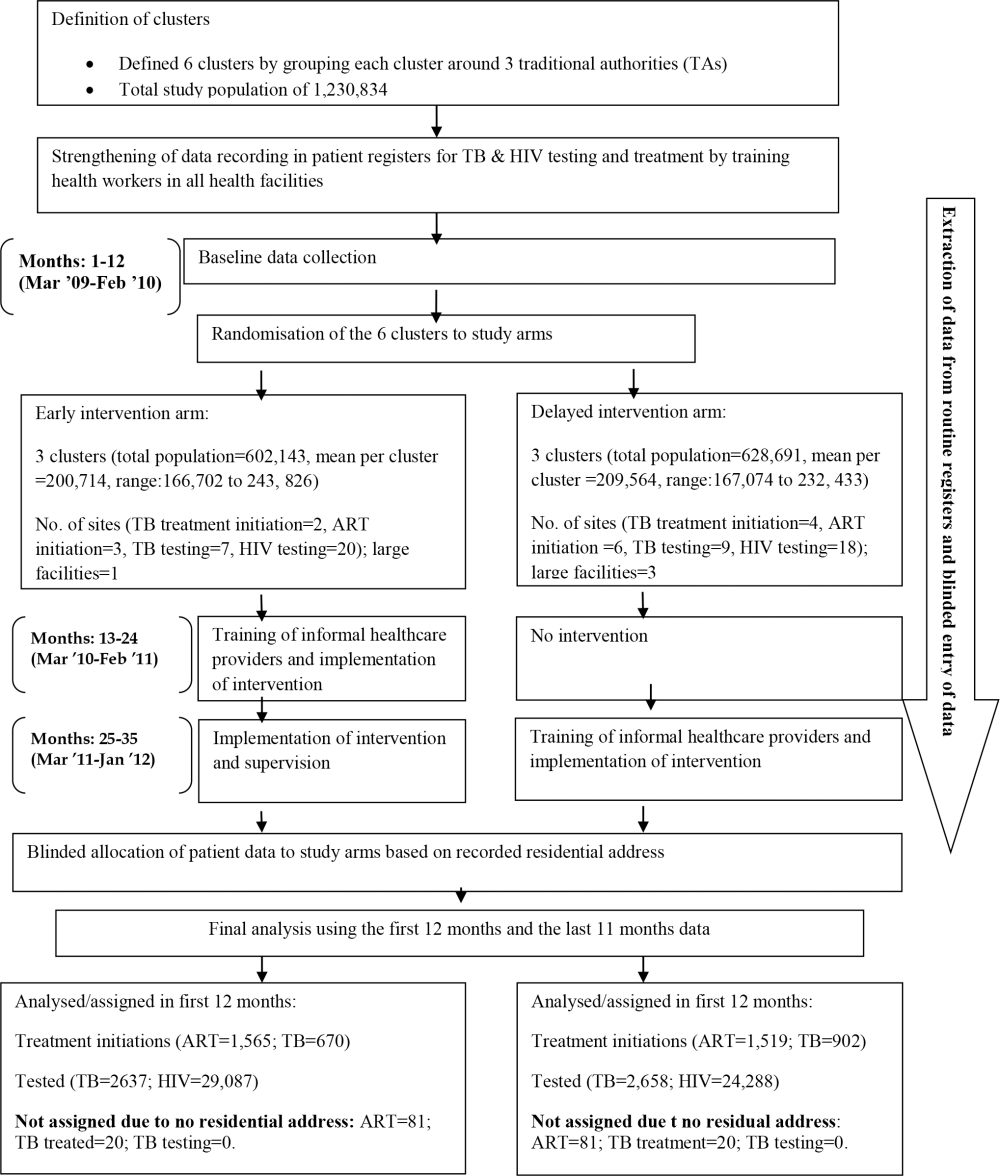
CONSORT participant flowchart.

The study populations in the different phases of the study are illustrated in the CONSORT Participant Flowchart (Fig 1).

**Fig 2:**
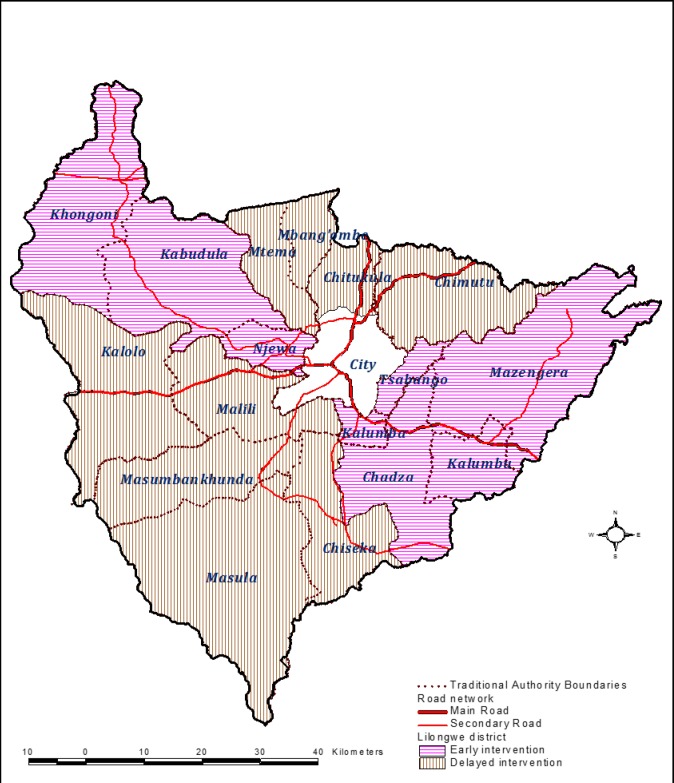
Map of Lilongwe District showing the distinction between urban and rural areas and the rural clusters allocated to the Early (pink) and Delayed (brown) arms of the trial.

Our previous research work on community engagement focused on urban and peri-urban Lilongwe where the involvement of unpaid storekeepers was effective in improving TB case detection [23]. As 85% of Malawi’s population lives in rural areas, we decided to focus the current Triage Plus study in the rural areas surrounding the city of Lilongwe, but within the borders of Lilongwe District (see Fig 2). The prevalence of infection with *Mycobacterium tuberculosis* is generally low in rural compared to the urban areas of Malawi [24]. To obtain an adequate number of TB cases in the study areas, large clusters were defined by grouping the villages covered by chiefs, officially known as Traditional Authorities (TAs). In total, 6 clusters were defined, and divided into two study arms: 3 clusters received the intervention in the first 12 months of the trial (Early) and 3 clusters received the intervention in the remaining 11 months of the trial (Delayed). Urban areas of the selected TAs were excluded. Each cluster was expected to include at least 160,000 people based on the previous census conducted in 2008 [25]. The average population sizes per cluster were 200,714 (ranged from 166,702 to 243,826) in the Early arm and 209,564 (ranged from 167,074 to 232,433) in the Delayed arm.

### The phased implementation and study design

From baseline, data for the study outcome measures were collected over the one year period before the training of informal providers. During this time all health facilities in Lilongwe District received additional training on how to complete existing routine TB and HIV testing and treatment registers, including accurate recording of residential address (see also under Data Collection below).

During the early design phase of the trial we considered whether the intervention should be implemented in the intervention clusters only with the other clusters remaining as control (non-intervention) clusters for a year. However, we were concerned about withholding the intervention from the communities in the control clusters, so it was decided that (as is becoming increasingly common practice in step-wedge study designs) the intervention would be implemented in the control clusters after one year.

**Fig 3:**
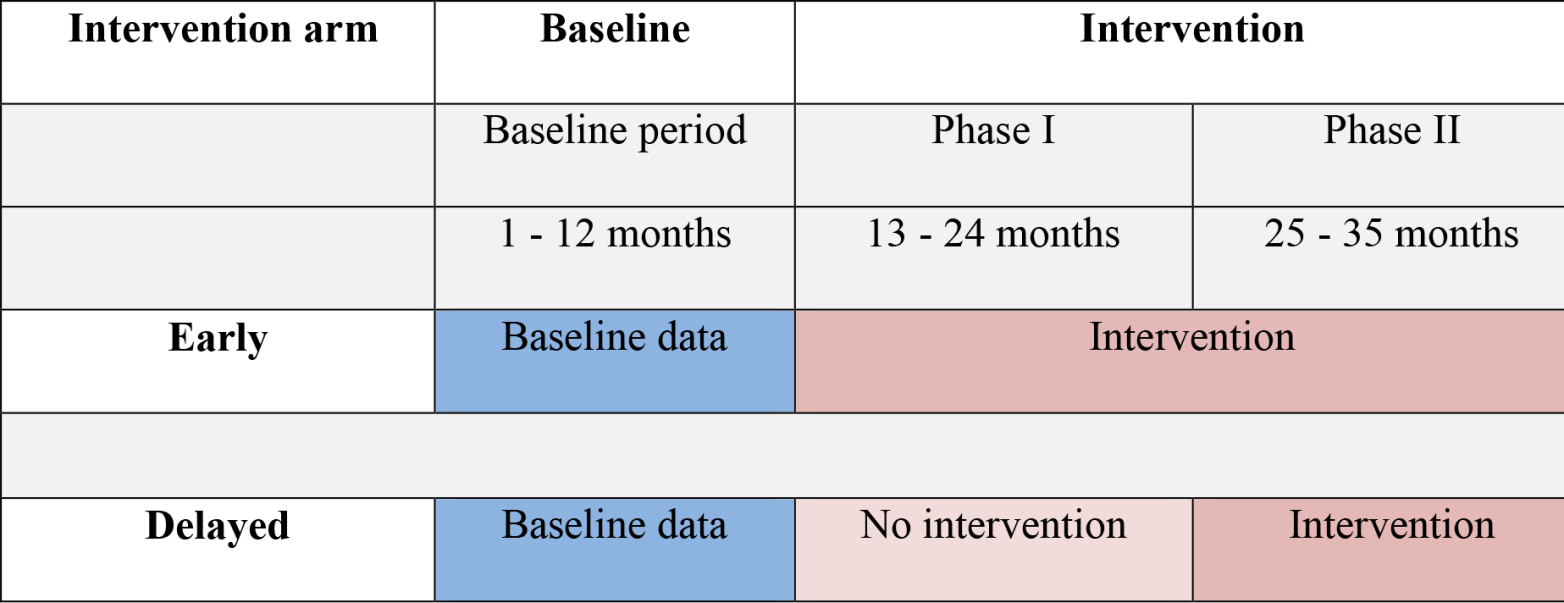
Triage Plus study design: Phased, pair-matched, parallel cluster design used to randomise study clusters to Early and Delayed arms. The blue colour represents the baseline period and the dark pink represents when the intervention package was implemented in the respective arms and the light pink represents study period when no intervention was implemented in the Delayed arm.

The three Early clusters therefore received the intervention for 12 months after the baseline data collection phase and the three Delayed clusters continued as controls. The Delayed arm then received the full intervention package for the next 11 months and the Early arm clusters continued to receive the intervention. Scheduling of the initiation of the intervention in each of the clusters was decided randomly with each selected cluster receiving the full training intervention before moving to the subsequent clusters. In all clusters arms, data collection continued for the entire trial period. Fig 3 summarises the phased, pair-matched, parallel cluster design used in the Triage Plus study.

### Engagement of informal healthcare providers

Recognising that Malawi has a serious shortage of formally trained health care workers, we did not want to add tasks to the workload of health care personnel who were already linked to the formal healthcare system. We therefore set the following eligibility criteria for engaging informal healthcare providers: a likely access point for poor and vulnerable people seeking TB and/or HIV services; physically established within the study area; embedded in existing community structures such as village health committees; not formally trained in clinical disciplines or public health, and not funded through the government or by international non-governmental organisations (iNGO’s). The informal health care providers who met the eligibility criteria included store-keepers, patient support groups (including youth groups), faith-based organisations or volunteers from local non-governmental organisations (lNGOs) or community based organisations (CBOs), village health committees, and herbalists. Thus their characteristics in terms of occupation and education level varied across all the study clusters and study arms.

### The intervention package

The intervention package consisted of training of informal health care providers to recognise individuals with symptoms suggestive of TB and HIV disease, encourage individuals with suggestive symptoms to attend a local health facility for diagnosis and clinical management, assist in TB sputum specimen collection, and conduct TB and HIV community awareness meetings. The informal providers could opt to implement one or more of the above elements according to their capacity. To ensure the informal health care providers received the necessary support to implement the intervention, health surveillance assistants were also trained, and they acted as a link between the informal health care providers and the public health facilities and provided supplies for sputum collection. They also provided supportive supervision to the informal healthcare providers, visiting at least once every quarter. In addition, all local leaders in the intervention areas were sensitised to the TB and HIV issues covered during the training of informal providers. This sensitisation of local leaders enabled the informal healthcare providers’ TB and HIV activities to be accepted in the community. The local leaders also provided support in arranging community meetings.

Intervention training materials and guidelines were developed to assist informal healthcare providers in implementing specific tasks. At community awareness meetings, the health messages included information on the signs and symptoms of TB, HIV and AIDS and emphasized that treatment is provided free in local primary care facilities. TB sputum smears were collected in the community by some informal healthcare providers and sent to the nearest primary care facility for microscopy. Results were fed back to the informal health care providers by local health surveillance assistants. Necessary precaution measures were taken to minimise TB infection during sputum collection by informal providers at community level. The measures included adequate training, sputum collection in the open air and use of appropriate sputum collection containers.

In total 1200 informal providers were trained during the study period. In the Early Clusters, the number of informal healthcare providers per cluster was proportional to the population size of the clusters with larger clusters having more informal healthcare providers. At this stage of the study, an average of 300 informal providers were trained per cluster. In the Delayed Clusters we decided to reduce the training intensity because of concerns about cost, so 100 informal providers were trained per cluster. Within each cluster (both Early and Delayed), the informal providers worked within a group of villages led by a group village headman (GVH). A minimum of one provider worked within each group of villages. Health surveillance assistants undertook supportive supervision to the informal healthcare providers, visiting at least once every quarter.

### Randomization and blinding

Before randomization, the 6 clusters were pair-matched according to population sizes and their proximity to the urban areas. Our experience at the outset of the study was that many rural residents seek care for serious conditions from health facilities within urban Lilongwe. These urban facilities have strong reputations as referral centres with more resources and expertise than rural facilities. We predicted, therefore, that some rural residents might be sensitized to the possibility of a TB or HIV diagnosis as a result of the Triage Plus intervention, but still decide to seek care from testing and treatment facilities within the city. All possible matched-pair combinations for the six clusters were considered. Using the data for the matching factors, “proximity” statistics were computed for each of these possible combinations based on squared Euclidean distances–that is, the extent to which the clusters differed for each possible matched-pair combination was estimated. The six combinations that produced the lowest (i.e. best match) “dissimilarity” statistics were identified and one of these selected at random for use in the study.

For this selected matched-pair combination, each of the six clusters was allocated a random number using the RAND() function in Excel. Then, for each cluster pair separately, the cluster with the smallest random number was assigned to arm A and the other to arm B. Two pieces of paper were then labeled “A” and “B”, placed in separate opaque envelopes of equal size and appearance, and sealed. One of the key stakeholders not involved in the randomisation process was asked to pick one envelope after the two envelopes were swapped several times. The code in the selected envelope was designated to be Early and the other to be Delayed.

The intervention was implemented at community level. Therefore it was not possible to conceal either the allocation of the intervention from the communities or from the research team involved in the study. However, at the analysis stage, allocation of patient data to specific clusters, based on their residential address, was conducted by an independent person who had no knowledge of the relationship between the addresses and the clusters (see below).

### Data collection

Because of our experience that many rural residents seek care for serious conditions from health facilities within urban Lilongwe (see above), we decided to collect data from routine testing and treatment registers in all health facilities in all of Lilongwe District; both rural and urban, but to allocate patients to the trial clusters on the basis of their residential address as recorded in the registers. This “intention-to-treat” principle had the additional benefit of minimizing possible bias. When patients residing in the Early clusters sought care in the Delayed clusters they would be assigned to the Early clusters if this was indicated by their residential address. The same would apply in reverse to patients with residential addresses in Delayed clusters who sought care in Early clusters.

The exception to this rule of allocation by residential address was the HIV testing data. As this is a confidential service, no residential addresses are recorded, so HIV testing data were allocated to clusters based on the location of the facility offering the testing service. In addition to this exception, data on HIV testing in some rural testing centres was incomplete in the second year of the trial and therefore the analysis of HIV testing data was limited to the first year.

“It is possible that the disparity in the distribution of health facilities offering TB and ART treatment initiation and testing services may have affected service access rates within the study arms (Fig 1), so the number of sites offering testing and treatment initiations were documented in the early and delayed clusters to show the distribution of such facilities between the two study arms (Fig 1). This permitted adjustment for any such imbalance by including this as a covariate in all models used when assessing the effectiveness of the intervention. Reporting the number of health facilities in the urban areas which were not part of the study areas was considered to give an indication of alternative sources of care for TB treatment, TB testing, ART initiation, and HIV testing of patients in the study areas but as these data were not expected to have any value as a potential covariate, they were not documented during the study period and so are not now available.”

The training that took place in the preparatory 12 months of the study of all health workers who were involved in recording data in the testing and treatment registers, was supplemented by regular supervisory visits and training during the 23 months of the trial.

Digital cameras were used to capture images of the testing and treatment registers and all TB and HIV treatment and diagnosis cases were abstracted from the electronic digital images and directly entered into an Excel database. Patient names and ages were initially used to identify duplicate entries but then removed from the database. The individual cases were then assigned to the respective clusters using their residential address which was indicated in the patient registers at the health facilities. Baseline data collection was conducted between March 2009 and February 2010 and data collection during the intervention period in both Early and Delayed clusters was conducted between March 2010 and January 2012.

### Outcome measures

The primary outcome measure for each of the two diseases was the cumulative count of patients initiating TB and HIV treatment per 10,000 adults aged 12 years and above each month over the study period. Secondary outcome measures included TB and HIV testing rates.

### Statistical analysis

With only six large clusters defined from the study areas, the sample size calculation to achieve adequate statistical power was based on simulation studies that were conducted using a repeated measurement design [26]. Please see the S1 Appendix for the simulation study results. Because there was one primary outcome measure for each of the two diseases (TB treatment initiation and ART treatment initiations), the simulation studies investigated the adequacy of statistical power for the low incidence events (such as TB treatment initiations) and the high incidence events (such as ART treatment initiations). Thus, baseline treatment initiation rates were used in the simulation process. We powered our study to be able to detect a 20% increase in the TB and ART treatment initiation rates as a result of the intervention. To achieve at least 80% power to detect a significant difference (two-sided p<0·05), with an estimated intra-cluster correlation in the range 0.00154 to 0.081, a mean baseline TB initiation rate of 15 per cluster per month, and a mean baseline ART treatment initiation of 70–80 per cluster per month, we needed at least 12 repeated measurements in each of the six clusters (three clusters per study arm). Thus, in the analysis we used monthly aggregated data over the trial period for all outcome measures.

To estimate the effectiveness of community engagement in increasing access to TB and HIV services, we first analysed the first 12 months data followed by the next 11 months data. The observed trends in access rates for TB and HIV services were summarised by calculating monthly and cumulative rates and we then used statistical modelling approaches to adjust for confounding effects. Estimation of the effectiveness of the intervention and investigation of heterogeneity in cluster level TB and ART treatment initiations rates as well as TB and HIV testing rates were investigated using marginal effects Poisson regression models with robust standard errors [27] as well as random effects models and adjusted for cluster level covariates [28]. Statistical adjustments were carried out using gender, baseline outcome measures and distribution of health facilities offering TB and HIV services. All analyses were carried out in Stata version 11.2. Only the results for the marginal effects modeling are presented here. Further details on the statistical analysis including results of the random effects modeling are available in the S1 Appendix.

## Results

All the phases of the study are illustrated in the CONSORT Participant Flowchart (Fig 1).

The 6 study clusters had a total population of 1,230,834; 49% in the Early arm clusters and 51% in the Delayed arm clusters, based on the 2008 National population and housing census. Mean cluster population sizes were 200,714 (ranged from 166,702 to 243, 826) in the Early arm and 209,564 (ranged from 167,074 to 232,433) in the Delayed arm (Fig 1 and Table 1). Overall, more sites offered HIV testing (n = 38) and treatment (n = 9) than TB testing (n = 16) and treatment (n = 6).

**Table 1:** Comparison of the baseline characteristics between Early intervention and Delayed intervention arms.

	Early Intervention	Delayed Intervention
**Demographic factors**	Estimate (SD^1^)	Estimate (SD)
Gender distribution for total TB cases starting treatment at baseline (% females)	49.3 (2.8)	41.7 (5.2)
Gender distribution for patients starting ART at baseline (% females)	62.1 (2.3)	61.0 (1.4)
Mean cluster catchment population at baseline	200,714	209,564
**Distribution of Health Facilities offering TB and HIV services**		
TB treatment initiation centres	2	4
TB testing centres	7	9
ART initiation centres	3	6
HIV testing centres	20	18
**Baseline monthly mean counts for TB and ART treatment initiation rates**^**2**^		
Mean (SD) TB cases starting treatment at baseline	15.0 (4.97)	17.3 (5.02)
Mean (SD) Smear-positive TB cases starting treatment at baseline	4.1 (0.85)	5.2 (1.20)
Mean (SD) ART cases starting treatment at baseline	32.97 (6.43)	33.2 (8.29)
**Baseline incidence rate ratios for TB and ART treatment initiation rates**		
TB cases starting treatment at baseline	111.93	123.75
Smear-positive TB cases starting treatment at baseline	31.39	37.06
ART cases starting treatment at baseline	245.95	236.68

^1^SD = Standard Deviation

^2^mean monthly counts with corresponding standard deviation in parentheses

The Early and Delayed clusters were well matched in terms of baseline monthly mean counts and unadjusted incidence rate ratios for TB and ART treatment initiation (Table 1). However there were fewer testing and treatment initiation facilities in the Early clusters than in the Delayed clusters (Table 1).

### Impact on TB testing and treatment initiations rates

After the first 12 months of the trial, the number of new presumptive TB patients accessing TB testing services was 2,637 in the early arm and 2,658 in the Delayed arm. Using the data from these 12 months, the effect of the intervention on increasing TB testing rates among presumptive TB cases before covariate adjustment was 4.2% (IRR = 1.042, 95% CI:0.8602–1.262; p = 0.676) and 15.2% (IRR = 1.152, 95%CI:1.009–1.359, p = 0.003) after adjusting for all covariates considered in the model (Table 2). The covariates adjusted for included baseline presumptive TB testing rates, number of testing sites in each cluster, gender distribution, time, and an interaction term between intervention status and time included as cluster-level covariates. All of the covariates were found to be significantly associated with TB testing rates (Table 2). In the second phase (11 months) of the trial, the unadjusted and adjusted incidence rate ratios were 1.016 (95% CI: 0.812–1.271; p = 0.892) and 0.659 (95% CI: 0.441–0.983; p = 0.023), suggesting that the intervention also improved the TB testing rates in the Delayed arm.

**Table 2:** Incidence rate ratios for intervention effects in access rates to TB diagnostic and treatment services over the 23 months of the intervention.

	Incidence rate ratio (95% CI)^1^	P value	Incidence rate ratio (95% CI)^1^	P value
The first 12 months of the trial	**TB testing**		**TB treatment initiation**	
Overall effect (unadjusted)	1.042 (0.860–1.262	0.676	0.824 (0.513–1,324)	0.676
Overall effect (adjusted)	1.152 (1.009–1.359)	0.003	1.18 (0.903–1.533)	0.114
*Covariates*:				
*Intervention Arm*^*2*^	*1*.*215 (1*.*071*, *1*.*379)*	*0*.*003*	*1*.*204 (0*.*957*, *1*.*515)*	*0*.*112*
*Baseline rate*^*3*^	*1*.*004 (1*.*004*, *1*.*004)*	*0*.*000*	*1*.*025 (1*.*003*, *1*.*048)*	*0*.*025*
*Month*^*4*^	*0*.*940 (0*.*906*, *0*.*975)*	*0*.*001*	*1*.*004 (0*.*978*, *1*.*031)*	*0*.*748*
*Gender*	*0*.*028 (0*.*028*, *0*.*029)*	*0*.*000*	*1*.*255 (0*.*306*, *5*.*140)*	*0*.*752*
*Interaction between trial arm and month*^*5*^	*0*.*948 (0*.*913*, *0*.*986)*	*0*.*013*	*0*.*981 (0*.*944*, *1*.*012)*	*0*.*335*
*Number of testing or treatment sites*^*6*^	*0*.*920 (0*.*919*, *0*.*921)*	*0*.*000*	*1*.*247 (1*.*096*, *1*.*419)*	*0*.*001*
The last 11 months of the trial				
Overall effect (unadjusted)	1.016 (0.812–1.271)	0.892	0.758 (0.445–1.290)	0.308
Overall effect (adjusted)	0.659 (0.441–0.983)	0.023	0.552 (0.397–0.767)	<0.001
*Covariates*:				
*Intervention Arm*^*2*^	*0*.*648 (0*.*445*,*0*.*943)*	*0*.*023*	*0*.*529 (0*.*387*, *0*.*722)*	*<0*.*001*
*Baseline rate*^*3*^	*0*.*993 (0*.*988*,*0*.*998)*	*0*.*008*	*1*.*038 (1*.*021*, *1*.*054)*	*<0*.*001*
*Month*^*4*^	*0*.*963 (0*.*959*,*0*.*967)*	*0*.*002*	*0*.*974 (0*.*959*, *0*.*989)*	*0*.*001*
*Gender*	*6*.*997 (2*.*421*,*20*.*231)*	*<0*.*001*	*0*.*320 (0*.*117*, *0*.*874)*	*0*.*026*
*Interaction between trial arm and month*^*5*^	*1*.*017 (0*.*992*, *1*.*043)*	*0*.*180*	*1*.*044 (1*.*027*, *1*.*062)*	*<0*.*001*
*Number of testing or treatment sites*^*6*^	*1*.*119 (0*.*757*,*0*.*876)*	*<0*.*001*	*1*.*269 (1*.*155*, *1*.*395)*	*<0*.*001*

^1^Incidence Rate Ratio (IRR) = rate in Early arm/rate in Delayed arm

^2^Intervention status (Early/Delayed)

^3^Baseline testing or treatment initiation rates

^4^Month of observation

^5^interaction between intervention status and measurement time

^6^number of testing or treatment sites.

**Fig 4:**
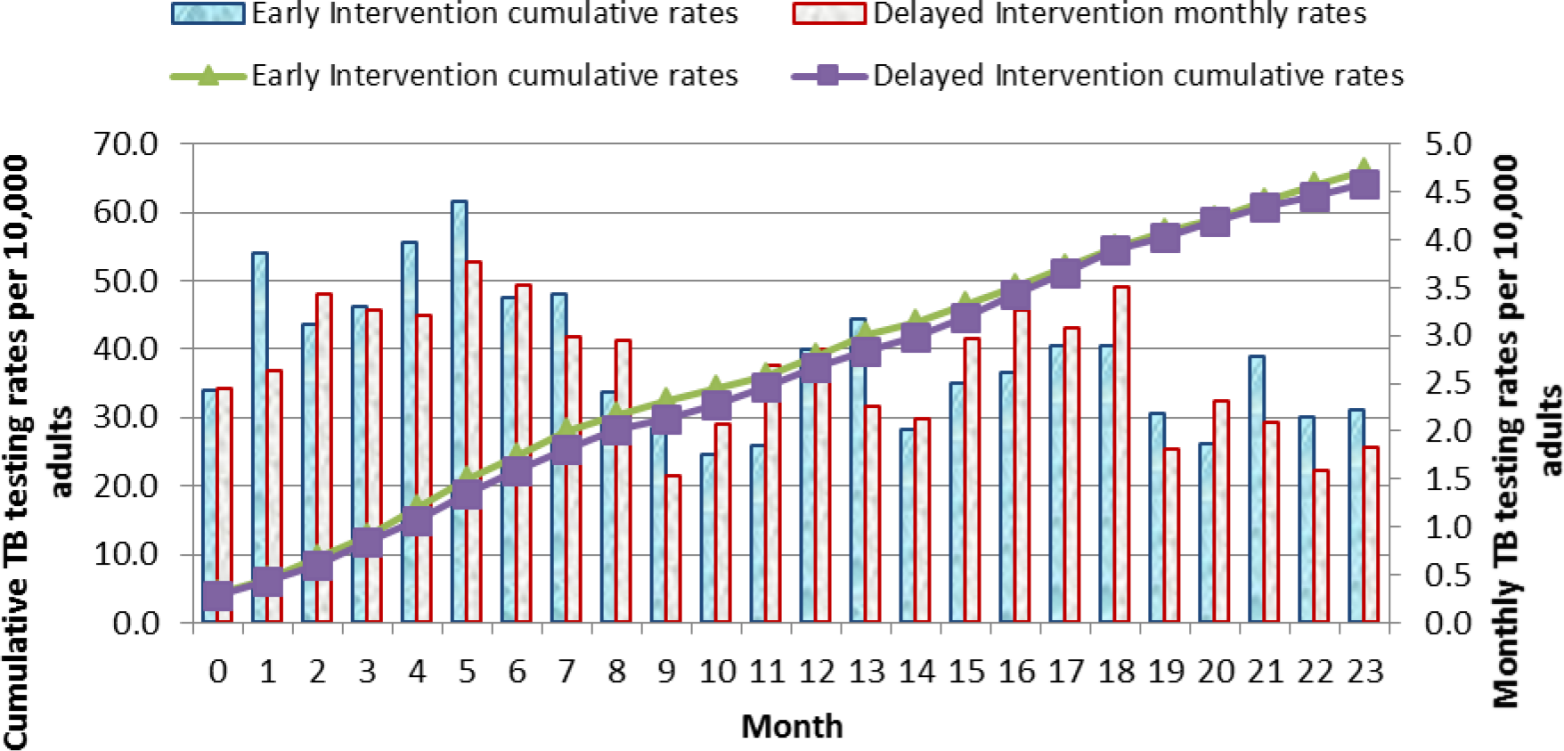
Monthly and cumulative TB testing rates per 10,000 adults: Results in individuals aged 12 years and above over the 23 months of the study are shown. The line graphs represent the cumulative TB testing rates. The green triangle line graph indicates the Early Intervention arm and the purple cubed line graph indicates the Delayed Intervention arm. The solid bar graphs represent the monthly TB testing rates per 1,000 adults for each month over the 23 months of the study. The blue bars represent the Early Intervention Arm while the grey bars represent the Delayed Intervention Arm.

Cumulative rates of TB cases accessing TB testing services over the 23 months study period showed more presumptive TB cases in the Early arm accessed TB testing services compared to the Delayed arm in the first 12 months and the trends reversed during the second phase of the intervention (Fig 4).

In analysis of the effect of the intervention on TB treatment initiation rates in the first 12 months, the estimated unadjusted incidence rate ratio was 0.824 (95% CI: 0.513–1.324 p = 0.424) and 1.18 (95% CI: 0.903–1.533; p = 0.114) after adjusting for all the covariates (including the interaction terms). This suggests that the intervention increased the likelihood of initiating treatment for TB cases in the Early arm by 18% compared to the Delayed arm, although this estimated effect size remained statistically non-significant (Table 2). Only baseline TB treatment initiation rates and the distribution of the health facilities initiating TB treatment remained statistically significant, indicating that a larger number of health facilities offering TB treatment initiations was also associated with an increase in TB treatment initiations, and that the increase in TB treatment initiations depended on the baseline TB treatment initiations in each cluster. In the next 11 months of the trial, the incidence rate ratio comparing the Early arm and Delayed arm was 0.758 (95% CI: 0.445–1.290; p = 0.308) before covariate adjustment and 0.552 (95% CI: 0.397–0.767; p<0.001) after adjustment suggesting that the intervention was effective in initiating more TB treatment in the Delayed arm when the intervention was scaled up in this arm. All the covariates considered in the model were significantly associated with TB treatment initiation rates (Table 2).

**Fig 5:**
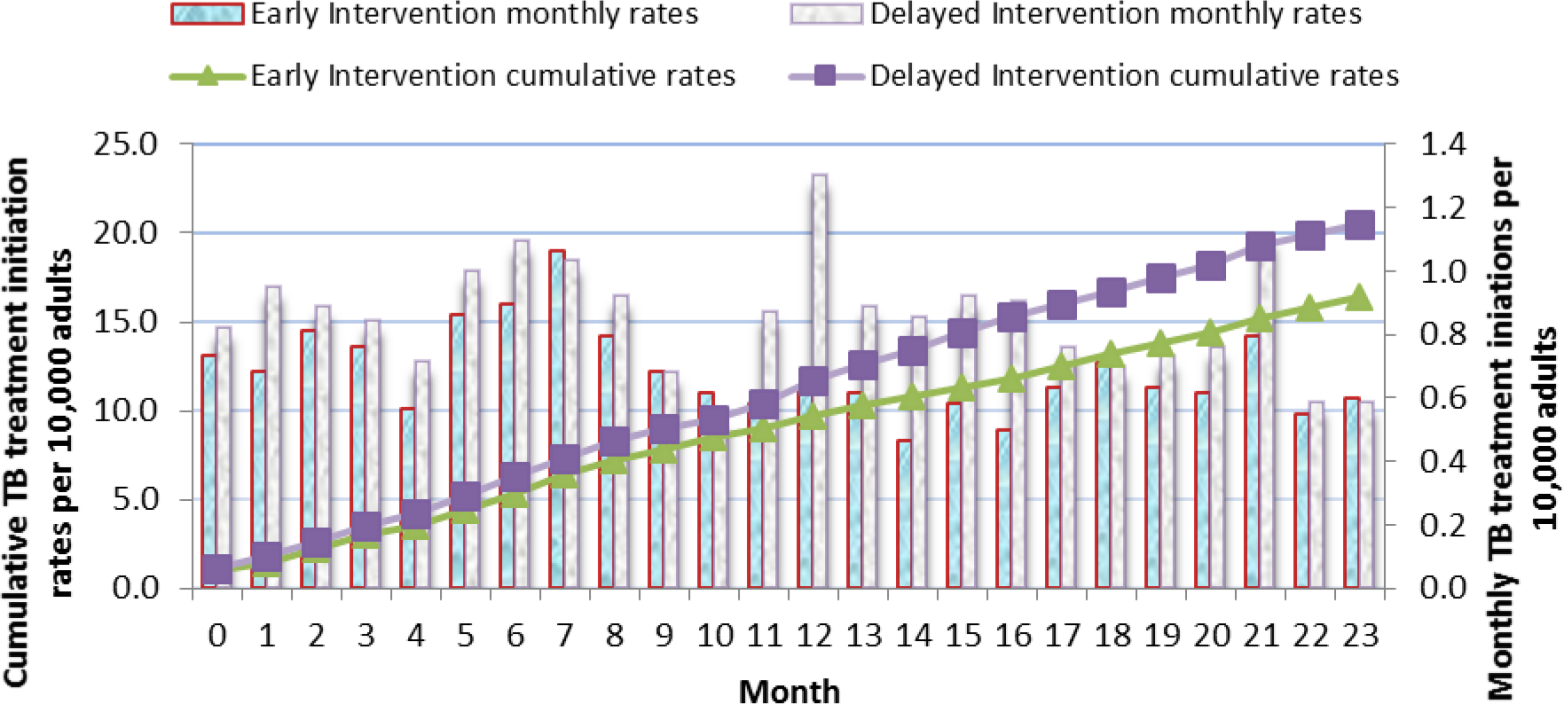
Monthly and cumulative TB treatment initiations rates per 10,000 adults: Results in individuals aged 12 years and above over the 23 months of the study are shown. The line graphs represent the cumulative TB treatment initiation rates. The green triangle line graph indicates the Early Intervention arm and the purple cubed line graph indicates the Delayed Intervention arm. The solid bar graphs represent the monthly TB treatment initiation rates per 1,000 adults for each month over the 23 months of the study. The blue bars represent the Early Intervention Arm while the grey bars represent the Delayed Intervention Arm.

Cumulative rates of TB treatment initiations over the 23 months study period showed fewer TB cases in the Early arm initiated on TB treatment compared to the Delayed arm (Fig 5).

### Impact on HIV testing and ART initiation rates

Data on HIV testing were only available for the first 12 months of the trial.

After the first 12 months of the trial, the number of clients accessing HIV testing was 29,087 in the Early arm and 24,288 in the Delayed arm. The incidence rate ratio during this time was 1.221 (95% CI: 0.810–1.839; p = 0.340) before covariate adjustment and 1.61 (95% CI:1.385–1.869, p<0.001) after adjustment (Table 3) suggesting an increase in testing as a result of the intervention. All covariates in the models, except baseline HIV testing measurements, were significantly associated with HIV testing rates. However, only the distribution of HIV testing sites (a proxy measure for geographical proximity) was significantly associated with increasing HIV testing rates by a substantial amount (IRR = 1.134, 95% CI: 1.079–1.193; p<0.001).

**Table 3:** Adjusted incidence rate ratios for intervention effects in access rates to HIV testing and treatment services over the 23 months of the intervention.

	Incidence rate ratio (95% CI)^1^	P value	Incidence rate ratio (95% CI)^1^	P value
The first 12 months of the trial	HIV testing uptake*		**ART treatment initiations**	
Overall effect (unadjusted)	1.221 (0.810–1.839)	0.340	1.128 (0.895–1.422)	0.308
Overall effect (adjusted)	1.61 (1.385–1.869)	<0.001	1.347 (1.00–1.694)	0.049
*Covariates*:				
*Intervention Arm*^*2*^	*1*.*626 (1*.*443*,*1*.*832)*	*<0*.*001*	*1*.*356 (1*.*003*, *1*.*833)*	*0*.*048*
*Baseline rate*^*3*^	*0*.*999 (0*.*999*,*0*.*9998)*	*0*.*016*	*1*.*003 (0*.*993*, *1*.*013)*	*0*.*567*
*Month*^*4*^	*0*.*949 (0*.*934*,*0*.*964)*	*<0*.*001*	*0*.*992 (0*.*962*, *1*.*024)*	*0*.*619*
*Gender*	*0*.*011 (0*.*003*,*0*.*039)*	*<0*.*001*	*0*.*319 (0*.*002*, *44*.*68)*	*0*.*650*
*Interaction between trial arm and month*^*5*^	*0*.*990 (0*.*960*,*1*.*020)*	*0*.*489*	*0*.*993 (0*.*949*, *1*.*040)*	*0*.*778*
*Number of testing or treatment sites*^*6*^	*1*.*134 (1*.*079*,*1*.*193)*	*<0*.*001*	*1*.*194 (1*.*069*, *1*.*333)*	*0*.*002*
The last 11 months of the trial*				
Overall effect (unadjusted)			0.799 (0.665–0.960)	0.016
Overall effect (adjusted)			0.924 (0.369–2.309	0.863
*Covariates*:				
*Intervention Arm*^*2*^			*0*.*927 (0*.*389*, *2*.*205)*	*0*.*863*
*Baseline rate*^*3*^			*0*.*994 (0*.*990*, *0*.*999)*	*0*.*011*
*Month*^*4*^			*1*.*08 (1*.*062*, *1*.*100)*	*<0*.*001*
*Gender*			*1*.*476 (0*.*128*,*16*.*960)*	*0*.*755*
*Interaction between trial arm and month*^*5*^			*0*.*997 (0*.*949*, *1*.*047)*	*0*.*898*
*Number of testing or treatment sites*^*6*^			*1*.*146 (1*.*093*, *1*.*201)*	*<0*.*001*

^1^Incidence Rate Ratio (IRR) = rate in Early arm/rate in Delayed arm

^2^Intervention status (Early/Delayed)

^3^Baseline testing or treatment initiation rates

^4^Month of observation

^5^interaction between intervention status and measurement time

^6^number of testing or treatment sites.

*no data were available on HIV testing uptake in the last 11 months

**Fig 6:**
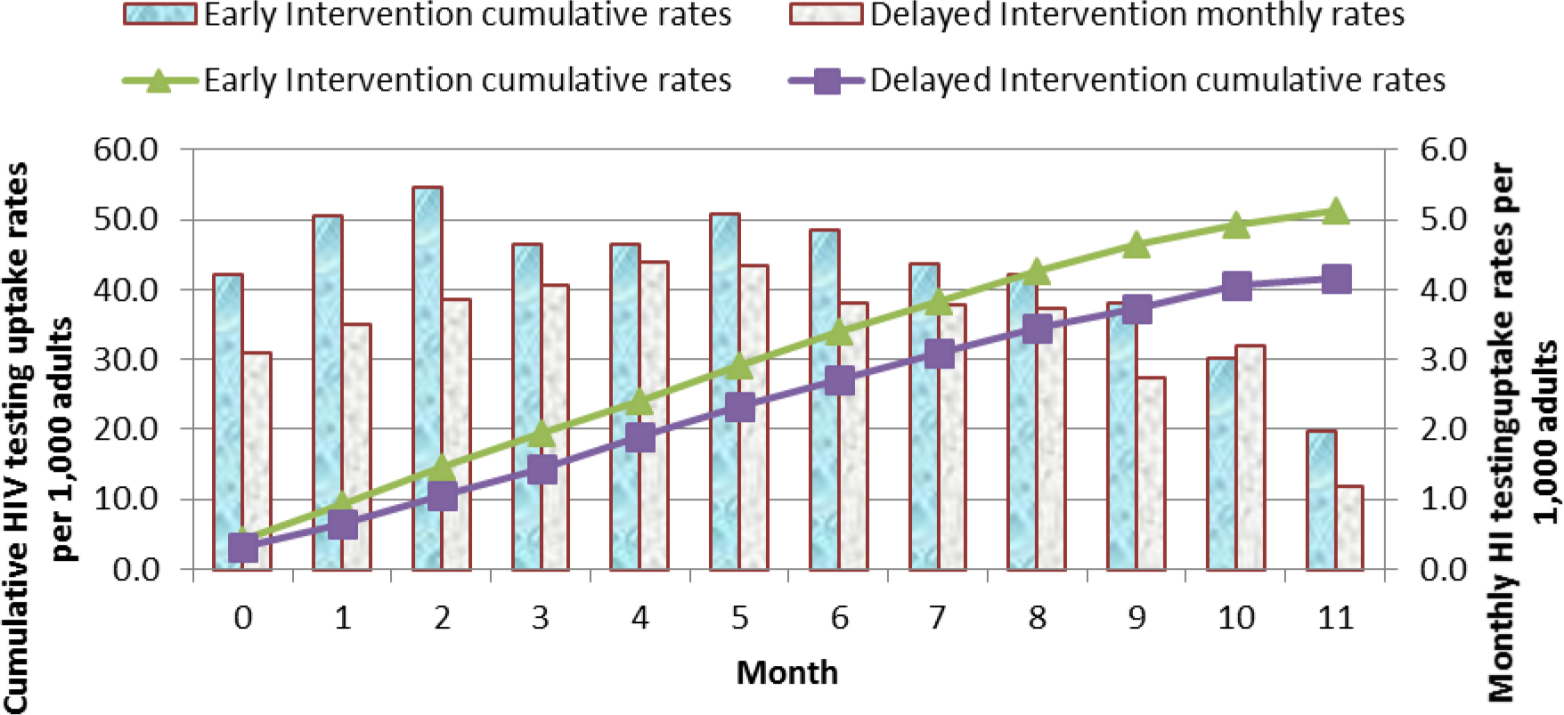
Monthly and cumulative HIV testing rates per 1,000 adults: Results in individuals aged 12 years and above over the first 11 months of the study before the scale up of the Delayed intervention arm are shown. The line graphs represent the cumulative HIV testing rates. The green triangle line graph indicates the Early Intervention arm and the purple cubed line graph indicates the Delayed Intervention arm. The solid bar graphs represent the monthly HIV testing rates per 1,000 adults for every month over the 11 months of the study. The blue bars represent the Early Intervention Arm while the grey bars represent the Delayed Intervention Arm.

Assessment of cumulative HIV testing rates for the first year based on non-adjusted data showed a greater increase in HIV testing rates in the Early arm compared to the Delayed arm (Fig 6).

The incidence rate ratio for the number of people starting ART in the first 12 months of the trial was 1.128, 95% CI: 0.895–1.422; p = 0.308) before covariate adjustment and 1.347 (95% CI:1.00–1.694; p = 0.049) after adjustment (Table 3). Distribution of health facilities initiating ART was significantly associated with ART initiation rates. A unit increase in number of health facilities was associated with a 19.4% increase in the number of ART patients starting treatment (IRR = 1.194, 95% CI: 1.069–1.333, p = 0.002). In the next 11 months of the trial, the incidence rate ratio was 0.799 (95% CI: 0.665–0.960, p = 0.016) before covariate adjustment and 0.924 (95% CI: 0.369–2.309, p = 0.924) after adjustment. This suggested that there were more ART patients initiating treatment in the Delayed arm when the intervention was scaled up in this arm (Table 3).

**Fig 7:**
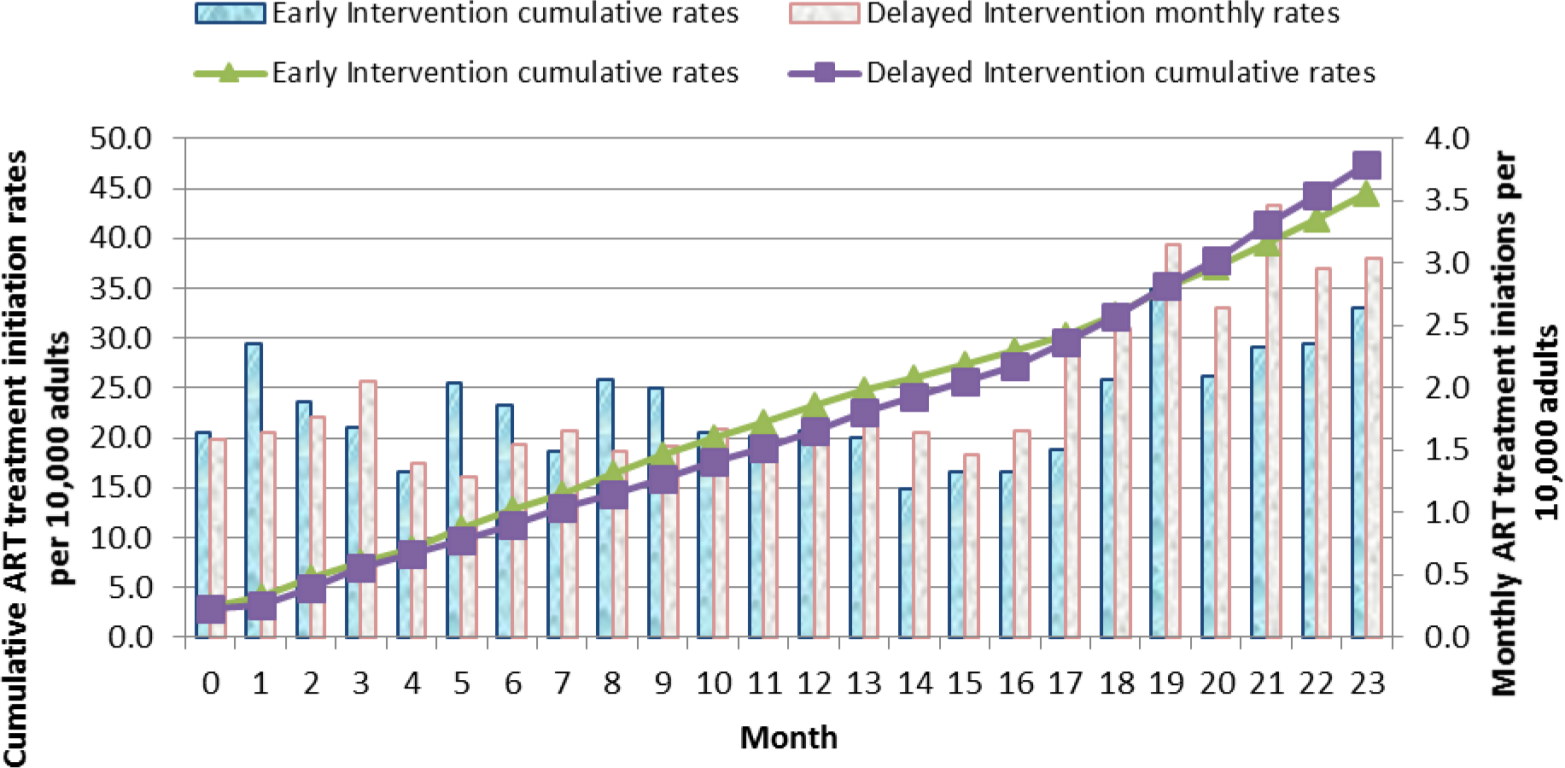
Monthly and cumulative ART treatment initiations rates per 10,000 adults: Results in individuals aged 12 years and above over the 23 months of the study before the scale up of the Delayed intervention arm are shown. The line graphs represent the cumulative ART initiation rates. The green triangle line graph indicates the Early Intervention arm and the purple cubed line graph indicates the Delayed Intervention arm. The solid bar graphs represent the monthly ART initiation rates per 1,000 adults for every month over the 23 months of the study. The blue bars represent the Early Intervention Arm while the grey bars represent the Delayed Intervention Arm.

Cumulative ART treatment initiation rates over the two years showed an increase in number of ART cases starting treatment in the Early arm in the first 12 months compared to the Delayed arm, and a catch up in ART initiation rates in the Delayed arm over the following 11 months (Fig 7).

## Discussion

Our results suggest that involvement of unpaid informal health care providers in rural Lilongwe significantly improved both HIV testing and TB testing rates. This involvement also increased ART treatment initiation rates. Previous experience with engaging close to community providers has shown similar results, but the providers have usually been remunerated in some way. A community based intervention in a rural district in KwaZulu-Natal, South Africa, involving paid community care workers to provide an integrated TB/HIV/PMTCT, showed improved rates for HIV testing (92% acceptance rates) and screening of sexually transmitted infections (32%) compared to sexually transmitted infections screening rates of 7% in control areas [29]. In Ethiopia, the engagement of extension health workers in the identification of presumptive TB cases has increased, and in some cases doubled TB case-detection [30]. Our group was the first to suggest the effectiveness of unpaid informal providers (in this case storekeepers alone) in TB case finding and treatment in peri-urban Lilongwe [23], and the current work extends this experience, for the first time, to HIV case finding and treatment.

Because we selected a range of informal providers according to a set of criteria rather than specific occupations, we believe that the intervention could be flexibly adapted to other contexts. We believe it would be relevant for similar settings in the region which have a comparable socio-demographic population profile, similar TB & HIV epidemiology and publically funded provision for both TB and HIV treatment and care. By allowing the informal health care providers to choose to implement one or more of the elements of the intervention to suit their existing community work, we believe that concerns regarding the sustainability of the interventions would be minimised. A separate paper on the cost-effectiveness of the intervention is being prepared.

By the end of the first 12 months, HIV testing and ART treatment initiation rates had increased by more than 61% and 34% respectively in the Early arm compared to the Delayed arm followed by a non-significant reduction in ART treatment initiation in the next 11 months. Furthermore, the differences in cumulative ART treatment initiation rates increased over time, with higher rates in the Early arm than in the Delayed arm in the first 12 months which then reversed after scaling up the intervention to the Delayed arm. This major improvement in ART treatment initiation and HIV testing rates following intervention suggests that the intensification of the engagement of informal healthcare providers in the delivery of HIV services in such under-served rural areas could likely lead to improved service access and, therefore, reduce HIV related deaths and transmission [31].

We have further showed that involvement of unpaid informal health care providers in HIV and TB community awareness, disease recognition, support in sputum collection and referral significantly increased TB testing rates. Results from the first 12 months showed that the engagement of informal healthcare providers increased the number of presumptive TB cases accessing testing in the Early arm by 15% (p = 0.003) compared to the Delayed arm followed by a significant increase in the number of TB cases accessing testing in the Delayed arm after scaling up the intervention.

Despite the significant increase in TB testing rates observed in the first 12 months of the study, the increase in the number of TB cases starting treatment in the Early arm compared to the Delayed arm was not significant. This was an unexpected finding, for which we have considered a number of possible explanations.

One potential explanation was stigmatization. The TB and HIV sensitisation messages were given as a package, highlighting the association between TB and HIV with a view to promote diagnosis and eventual treatment initiations for the two diseases. This association can be a major source for stigmatisation and may affect timely presentation for TB treatment in high HIV prevalence areas [32]. In a community survey exploring attitudes to TB in South Africa the majority of the respondents (89%) cited fear of learning one's HIV-positive at health clinics and the belief that almost all TB patients are HIV positive and that only people who are HIV positive get TB were found to be the most important barrier for timely TB case detection [33]. However, the improved testing rates for both TB and HIV in the Early arm in the first 12 months suggests that stigmatization is not the explanation for the lack of effect of the intervention on TB treatment initiation.

A second possible explanation was a falling TB incidence over the time period of the study. Community interventions using participatory approaches have led to major reductions in latent TB incidence rates within a few years [15]. O’Donnell and colleagues observed a sustained reduction in the incidence of latent TB infection in a community based participatory intervention. Incidence of latent TB infection reduced by 50% from the baseline levels over a 3 year period. Using active case finding strategies in high-density residential suburbs in Harare, Corbett and colleagues observed a 40% reduction in smear- positive TB cases from the baseline at the end of 3 years [10]. However, TB incidence in our study could only have been reduced as a consequence of the intervention in the Early arm if TB treatment initiation had been significantly increased. If TB incidence was already falling in both the Early and Delayed arms as a result of background effectiveness of the TB programme, then the intervention should still have resulted in a detectable increase in TB treatment initiation.

Overall we believe that the most likely explanation relates to the extent to which TB testing and HIV testing are available in the rural areas. HIV testing was available at 20 sites in the Early arm and 18 sites in the Delayed arm. This compares with TB testing which was only available at 7 sites in the Early arm and 9 sites in the Delayed arm (see Fig 1). We believe that a more likely explanation for the lack of increase in TB treatment initiation is that people in this poor, rural area will have used substantial resources (including for travel) to access the infrequently available TB testing sites. Previous work from our group suggests that TB patients in peri-urban Lilongwe use between 2 and 5 times their monthly income in reaching a TB diagnosis and starting treatment [34]. By contrast, patients accessing the more frequently and locally available HIV testing facilities will have used a smaller percentage of their available resources. In support of this, work in Malawi suggests that patients accessing ART spend less than their monthly income in care-seeking (Namakhoma et al submitted). Furthermore, there is evidence that linkage between HIV testing and ART initiation is hindered when treatment initiation is less accessible [35]. Overall, therefore, we hypothesise that people who have accessed TB testing will frequently have depleted their resources to the extent that going on to access TB treatment initiation will have been beyond their means. By contrast, people who have accessed HIV testing will not have depleted their reserves to the same extent and will have found it easier to travel to, and access the HIV treatment initiation sites. Similar numbers of HIV and TB treatment initiation sites were available in both arms of the study: 3 and 2 respectively in the Early arm and 4 and 6 respectively in the Delayed arm.

Despite the strength provided by adoption of a cluster randomised design coupled with the multiple measurements within a limited cluster randomisation and the use of random effects modeling, the study had some inherent limitations.

First, although he use of repeated measurements might have addressed the statistical power issues in terms of having adequate degrees of freedom, the limited number of clusters used available within the study area may have compromised the balance of confounding factors that randomization seeks to achieve [36–38].

Such factors could have contributed to the variation in response rates between the clusters. In addition, the intervention was complex and diverse with many components, each of which would have impacted to different extents across the clusters, adding further to the effect heterogeneity observed. To take a specific example, storekeepers and herbalists were eligible informal health care providers; if such individuals were more reluctant to refer due to concerns about losing potential business, clusters with a high volume of such providers might provide (negatively) biased estimates of effect size. Such biases do not invalidate the core conclusion; this study provides strong evidence that a health system policy intervention of this type can impact very positively on TB and HIV case detection and treatment initiation rates in a rural setting. The biases do, however, mean that response rates will vary from context to context. The data that were collected in this study was insufficient to allow for detailed differentiation of the effects of the different components of the intervention.

Second, being a community intervention study, we could not achieve complete blinding to healthcare workers, research team and the communities involved in the study and this had the potential of having the intervention leaked to control areas leading to an apparent reduction in the impact of the intervention. Third, the evaluation process depended on routine data recorded in patient registers in health facilities; therefore, potentially inaccurately recorded patient address details were used to allocate patients to the study arms. The use of routine data may have contributed to biased estimates of the intervention effect for several reasons:

1. Patients who received treatment in urban areas while temporarily residing with relatives due to their illness may have been less likely to mention their actual place of residence and, therefore, may have been missed in our database, which only collected patients with rural addresses, leading to an underestimation of the number of TB or HIV cases in the rural areas. No specific direction of the bias could be determined as this was likely to occur on all study arms.
2. As the intervention improved the community's knowledge of TB and HIV and the relationship between the two, a certain proportion of patients may have either avoided seeking health care services for fear of being labeled HIV positive [33] or used false names and addresses in the course of seeking health care for fear of being identified and traced. This may have lowered the effect size of the intervention.

To ensure the scalability of the intervention by the national programme, implementation of the intervention was flexible, in terms of types of informal health care providers engaged and as well as the nature of activities implemented by them. In addition, by allowing the informal health care providers to choose specific intervention activities, which they were capable of doing or were already doing as part of their existing community work, concerns regarding the sustainability of the interventions were minimised.

However, as discussed above, the estimate of the intervention effect size could have been influenced by potential biases in the design and nature of intervention. In a complex intervention such as the one evaluated in this paper, the effects of the intervention components will vary, both within and between communities. The study could have collected data to allow for a detailed exploration of the interactions between the intervention components, but this would have risked deviating the trial too far from its pragmatic roots. Our previous work with grocery store providers had already demonstrated that focal engagement of one type of provider was beneficial [23]. Local policy makers have been looking for evidence about a broader intervention requiring less ground-work to differentiate providers, believing this to be a more easily implementable approach to large scale roll-out.

## Conclusions

The Triage Plus intervention showed that the engagement of unpaid informal providers in integrated TB and HIV services at community level increased access to TB and HIV testing as well as increased ART initiation in this rural context. However, for unpaid, informal providers to be effective in promoting increased TB treatment initiation, TB services need to be further decentralized so that both TB testing and treatment initiation sites are more locally accessible to the population.

## Supporting information

S1 Appendix Sample size determination using simulation and random effects modelling. (DOCX)

S2 Appendix Completed CONSORT 2010 checklist. (DOC)

S3 Appendix Trial protocol. (DOC)

S4 Appendix Trial data for ART initiation. (DTA)

S5 Appendix Trial data for TB treatment inititation. (DTA)

S6 Appendix Trial data for HIV testing. (DTA)
