# Supplementary material for: The effect of engaging unpaid informal providers on case detection and treatment initiation rates for TB and HIV in rural Malawi (Triage Plus): A cluster randomised health system intervention trial
Source: PLoS One. 2017 Sep 6;12(9):e0183312. doi: 10.1371/journal.pone.0183312 (PMC5587112; doi:10.1371/journal.pone.0183312)
Supplement: S1 Appendix — (DOCX) [file pone.0183312.s001.docx]

# Supplementary Material/Web appendix

# Sample size determination using Simulation and Random Effects Modelling for “The effect of engaging unpaid informal providers on case detection and treatment initiation rates for TB and HIV in rural Malawi (Triage Plus): a cluster randomised health system intervention trial “

**1.0 Sample size determination using simulation studies**

## Introduction

Data obtained from cluster randomised interventions are usually correlated; therefore, multilevel modelling approaches are recommended for analysis (Breslow and Clayton, 1993; Gelman and Hill, 2007). The maximum likelihood estimation methods commonly used in multilevel modelling are asymptotic, and these methods assume that the sample sizes will be large enough for accurate estimates. However, in randomised studies that use the cluster as the unit for treatment allocation, a common drawback is that the number of clusters available is usually limited (Murray, Varnell and Blitstein, 2004; Pals, Wiegand and Murray, 2011; Simwaka et al., 2012). Adding more clusters may in itself raise distinct challenges, ranging from the increased costs incurred by the addition of clusters, to problems of contamination (Torgerson, 2001; Borm et al., 2005) if the clusters are too small. Furthermore, the outcome of interest may have low prevalence or incidence necessitating larger clusters to achieve an adequate sample size in each cluster. However, it is an inescapable fact that having only a limited number of clusters raises questions about the statistical power as well as the accuracy of the effect size estimates and their standard errors obtained in such studies (Moineddin, Matheson and Glazier, 2007).

With the six clusters used in this study, there was a clear need for further investigations into the required design conditions necessary for achieving optimal statistical efficiency to guide the statistical analysis of the study.

## The Simulation study

Because the findings of the simulation studies were intended primarily to inform which analytical approach should be used for the actual data derived from the study, and as the primary outcome in this study was a count variable, Poisson generalised linear mixed models (GLMM) were used to assess statistical efficiencies under different design conditions. In particular, the relative efficiencies of Poisson GLMM regression methods were evaluated using likelihood estimation approaches in the estimation of intervention effect size. To run the simulations, the baseline dataset was used as a ‘training dataset’ to generate the random sample datasets to obtain the desired parameter specifications such as the ICC and mean response per group.

The study used only 3 clusters per arm, and the design involved the collection of repeated observations in each cluster over time. The simulation studies reported below generated cross-sectional panel data over a 12 month intervention period in line with the Triage Plus study. Thus, the maximum units of analysis used were 3 x 12 = 36 cluster-months per intervention arm (varying from 3x2 to 3x12 cluster-months for 2 to 12 repeated measurement occasions respectively). These simulations were used to investigate the likely statistical power and efficiencies of the study design and then used in the analysis to assess the effectiveness of community engagement in improving access to TB and HIV services. The simulations were then extended to involve other likely scenarios such as increased ICCs for comparison.

### Objectives

The objective of the simulation studies were to:

1. determine the optimal design conditions such as number of repeated measurement time points, disease incidence and effect size to achieve adequate statistical power of at least 80% in a study with three clusters per arm.

###

### Simulation procedures

**Generation of data**

Simulations were constructed with repeated measurements in each cluster using desired parameter specifications. The distributions and parameters specified included the mean response per cluster and the desired effect size. Since the simulations represented a cluster randomised trial design with repeated measurements, covariate correlation structures, which summarised a correlation between clusters, and repeated measurements within clusters were also specified (Arnold et al., 2011). More specifically, the parameter specifications in the statistical models were estimated from the real data obtained from the baseline data for the Triage Plus study and used to generate the random sample data sets by fitting the model without covariates to obtain the model intercept and the between and within cluster variance estimates for the ICC. Because there were four different outcome measures with varying between and within cluster correlations, we run the simulations using ICCs of 0.00154, 0.081, 0.321 and 0.699. The ICCs of 0.00154 and 0.081 corresponded to low intracluster correlations and the others corresponded to high intracluster correlations.

To generate the random outcome, a generalised linear mixed model (GLMM) framework was used to account for the correlations resulting from the randomised cluster design as well as the repeated measurements from each cluster. Given $y_{ij}$ is an outcome response at repeated measurement occasion $i$ ($i=2,\ldots, T$) indexing time points within clusters, $(j=1,\ldots,N$) indexing clusters, which is conditionally Poisson and distributed with mean $\mu_{ij}$, the statistical model used in the simulations is

$$y_{ij}|x_{ij},u_{j}, u_{ij}\sim Poisson \left( \mu_{ij} \right)$$

$log \mu_{ij}=\log\left( n_{ij} \right)+ \beta_{0}+\beta_{1}x_{ij}+u_{ij}+ u_{j}$ (1)

$${u_{ij},u}_{j}\sim N(0, \sigma^{2})$$

where:

$y_{ij}$ denotes the number of events measured at time point $i$ in cluster $j$ and is conditionally Poisson distributed. The time points were varied from 2 to 12.

$\mu_{ij}$is the mean count for cluster $j$ at time point $i$,

$\log\left( n_{ij} \right)$ is an offset with a constant coefficient of 1,

$\beta_{0}$ is the mean number of events in the control arm ,

$\beta_{1}$ is the estimated intervention effect comparing intervention clusters and control clusters ,

$x_{ij}$ is a covariate for intervention status (i.e. treated or not),

$u_{j}$ is a random effect for individual cluster $j$ to model between cluster variation

$u_{ij}$ is a random effect at time point $i$ and cluster $j$ to model both extra-Poisson and variability within clusters due to repeated measurements.

The random effects are assumed to be normally distributed with a mean of zero and a known standard deviation.

The intracluster correlations resulting from these random effects affect statistical power as well as the accuracy of the parameter estimates (Moineddin, Matheson and Glazier, 2007; Parker, Evangelou and Eaton, 2005). The ICC is based on the relationship of the between-to-within-cluster variance and is given by:

$$ICC=\rho=\sigma_{b}^{2}/(\sigma_{b}^{2}+\sigma_{w}^{2})$$

where:

$\sigma_{b}^{2}$ is the variance of random intercept that represents the between-cluster variation

$\sigma_{w}^{2}$ is the within-cluster component of the variance (Parker, Evangelou and Eaton, 2005).

According to Nakagawa and Schielzeth (2010), the within cluster variance $\sigma_{w}^{2}$ on the log link scale in Poisson mixed effects models is given as:

$$\sigma_{w}^{2}=ln(\frac{1}{\exp\left( \beta_{0} \right)}+1)$$

Thus, to generate the outcome variable $y_{ij}$, the fixed effects parameters and the standard deviations for between and within clusters, $\sigma_{b}$ and $\sigma_{w}$, were specified. The intervention effect size $\beta_{1}$ was specified based on pre-determined intervention effects of 20%.

To estimate the remaining 3 parameters ($\beta_{0}, \sigma_{b}, {\mathrm{and} \sigma}_{w})$, baseline datasets for the Triage Plus study was used to estimates the parameters by fitting a model without covariates (Arnold et al., 2011) as

$$y_{ij}|x_{ij},u_{j}, u_{ij}\sim Poisson \left( \mu_{ij} \right)$$

$log \mu_{ij}=\log\left( n_{ij} \right)+ \beta_{0}+u_{ij}+ u_{j}$ (2)

Once the desired parameters were estimated, 12 repeated measurements, datasets and random effects were then generated. Cluster level data was created by expanding the data sets to the required number of clusters, and corresponding random effects were generated for each cluster. The outcome response $y_{ij}$was then simulated using models (1), and Poison mixed effects regression models (1) were fitted to the data set with indicators contrasting the intervention or control specified in the model. Regressions coefficients were saved for further analysis.

**Design conditions investigated**

In the simulation of data sets, the following factors were allowed to vary: number of clusters per arm, effect size in the intervention arm, number of repeated measurement points and incidence rate ratios as follows:

1. The number of clusters per arm were set at 3.
2. The intervention effect sizes were based on incidence rate ratio (IRR) and specified as 1.1, 1.2, 1.3, 1.4, 1.5, 1.6 and 1.8 by varying the regression coefficient of the variable contrasting intervention and control (the effect size of 1.2 or 20% increase in uptake represented the desired effect of the intervention in the Triage Plus study). These intervention effects were fixed during each of the simulations which were then compared to the estimated intervention effects after 1000 simulations.
3. As the risk of encountering estimation problems increases as the number of data collection (time) points increases (due to the reduced number of potential observations at each fixed time) (Tutz and Kauermann, 2003), the intervention’s effect was evaluated at time points for repeated outcome measurement, set at 2, 3, 4, 6 and 12. Basic baseline and post-intervention study design were represented by 2 measurement points: 12 measurement points represent the desired 12 month period for the project implementation used in Triage Plus.
4. Because of the differences in the incidence of TB and ART treatment initiations which are likely to affect the statistical power required to detect a significant effect of the intervention and the accuracy of the parameter estimates (Raudenbush and Liu, 2000; Moineddin, Matheson and Glazier, 2007), statistical power were also assessed by varying disease incidence based on the actual data derived from baseline data set of the Triage Plus study (Dean et al. 2004; Burton et al., 2006, Arnold et al., 2011). This was done by varying the mean number of events (new or incident cases of TB and HIV treatment initiation rates) in the control clusters (Delayed arm), which corresponded to the intercept of the regression model.

**Quantities of interest in each simulation**

The parameters of interest stored after fitting Poisson mixed effects regression models included fixed effects regression coefficients ($\beta_{i}$) assumed to be constant across time points and their associated simulation standard errors (${SE}_{i}$). The mean estimate $\overline{\beta}$ over the $N$ simulations conducted were obtained under each of the conditions investigated as a measure of the true estimate of interest (in our case, N=1000).

The mean estimates derived from the regression coefficients were then exponentiated to obtain incidence rate ratios. To show the levels of uncertainty around these estimates, empirical standard errors (calculated as the standard deviation of the parameter estimates over all the simulations) and the mean of the within simulation standard errors of the parameter estimates were calculated according to Burton et al. (2006). To confirm that the simulations were correctly done, a simulation of a Null case (H0: B1=0) was carried out so that the distribution of the *p*-values was uniform.

### Assessment of statistical power estimation for different design conditions

The parameter estimates of the 1000 saved simulations completed under each of the studied design conditions were used to calculate test statistics (z test) and p-values as described by Arnold et al (2011). Statistical power for each design condition was then estimated as a proportion of the total observations for which the p-value is less than the conventional significance level of 5% (α = 0.05).

### Assessing performance of the different statistical methods

As statistical efficiency may vary with design conditions, statistical efficiency under different design conditions using various performance measures was assessed (Maas & Hox, 2005; Moineddin, Matheson & Glazier, 2007; Burton et al., 2006; Arnold et al., 2011; Collins, Schafer and Kam, 2001). Performance measures used to evaluate efficiencies in parameter estimation included bias, mean square error, the average length of the 95% confidence intervals of parameters estimates and the coverage of the 95% confidence intervals as applied in similar studies. The simulated results were compared against the true values used to generate the data. However, for this Appendix we only report results for the convergence (monitored using stata's built-in procedures) and power estimates.

## Results

### Convergence

The convergence rate was determined as the proportion of simulations that converged. The convergence properties were investigated under the different scenarios. Overall, model convergence rates (percentages converged) were high in all scenarios investigated and varied from 97.5% to 100%. Unsurprisingly, therefore, there was no clear pattern in convergence rates after varying the number of repeated measurements, the effect sizes and/or incidence of the disease conditions confirming the findings of previous studies (Moineddin, Matheson and Glazier, 2007).

###

### Power estimation

Statistical power determination was calculated as the proportion of the total observations for which the observed p value was ≤ 0.05 since the simulation runs that fail to identify statistically significant differences are technically Type II errors since in the simulation β_1_ ≠0 was assumed (Arnold et al, 2011). We took a power of at least 80% to be adequate (Cohen, 1992).

#### Power estimates when the ICC is set 0.00154

With 3 clusters in the Triage Plus study, the simulation studies indicated that the Triage Plus Trial would have had only 24% statistical power to detect a 20% improvement in the detection and initiation of TB treatment if only one cross sectional dataset at the endline was to be used. However, statistical power would have increased with increasing effect sizes and the number of repeated measurement times. Adequate statistical power of 80% would be achieved with the desired effect size of 20% if there were 12 repeated measurement times. However, with an effect size as low as 10%, inadequate power is achieved even with 12 repeated measurement times (37% power at 12 measurements) for low incidence events (see Figure 1). Figure 2 shows power estimates for higher incidence events. When the ICCs were varied, adequate power estimates could not be achieved with high ICCs even with increased number of repeated measurement time points for outcome measures with a maximum of 70 cases per month (Table 1).

Figure 1 **Power curves:**

The graphs show the relationship between power and the number of repeated measurement time points at different effect sizes (10%, 20%, 30%, 40%, 50% and 60%) in the 3 clusters per arm design using 1000 simulated datasets, when incidence is low and ICC was 0.00154.

Figure 2 **Power curves:**

The graphs show the relationship between power and the number of repeated measurement time points at different effect sizes (10%, 20%, 40% and 60%) in the 3 clusters per arm design using 1000 simulated datasets, when incidence is high and ICC was 0.00154.

| Table 1: Power estimates for different effect sizes (20%, 40%, and 60%) and different ICCs (0.081, 0.321, and 0.699) | | | | | | | | | | |
| --- | --- | --- | --- | --- | --- | --- | --- | --- | --- | --- |
|  |  | 0.081 | | | 0.321 | | | 0.699 | | |
|  | Effect | 1.2 | 1.4 | 1.6 | 1.2 | 1.4 | 1.6 | 1.2 | 1.4 | 1.6 |
| 3 clusters | Time |  |  |  |  |  |  |  |  |  |
|  | 2 | 42.8 | 79.1 | 95.3 | 22.8 | 39.2 | 48.3 | 15.8 | 20.4 | 21.3 |
|  | 3 | 46.5 | 84.3 | 97.0 | 24.4 | 37.9 | 49.6 | 21.1 | 19.7 | 24.3 |
|  | 4 | 51.9 | 87.9 | 98.1 | 23.7 | 35.7 | 53.0 | 20.5 | 20.5 | 21.8 |
|  | 6 | 51.3 | 89.8 | 98.4 | 23.3 | 35.2 | 54.3 | 18.6 | 23.9 | 21.4 |
|  | 12 | 51.0 | 87.7 | 98.4 | 23.1 | 38.4 | 51.5 | 18.8 | 18.4 | 21.6 |
| 4 clusters |  |  |  |  |  |  |  |  |  |  |
|  | 2 | 49.2 | 85.5 | 96.8 | 21.5 | 34.3 | 56.6 | 14.9 | 17.0 | 17.7 |
|  | 3 | 49.6 | 91.0 | 99.7 | 21.0 | 35.0 | 56.2 | 16.4 | 17.2 | 19.6 |
|  | 4 | 55.5 | 93.1 | 99.3 | 19.9 | 37.0 | 53.0 | 15.3 | 16.1 | 17.3 |
|  | 6 | 56.9 | 91.8 | 99.8 | 23.8 | 37.9 | 55.2 | 14.5 | 17.5 | 19.7 |
|  | 12 | 56.9 | 94.5 | 99.7 | 22.9 | 38.4 | 54.7 | 14.2 | 18.0 | 17.6 |
| 5 Clusters |  |  |  |  |  |  |  |  |  |  |
|  | 2 | 52.4 | 91.8 | 98.1 | 19.6 | 40.7 | 56.6 | 13.5 | 13.4 | 16.4 |
|  | 3 | 60.8 | 94.3 | 99.7 | 19.7 | 43.2 | 59.4 | 11.7 | 14.8 | 15.9 |
|  | 4 | 58.8 | 96.4 | 99.9 | 22.0 | 35.1 | 58.0 | 14.4 | 14.4 | 18.1 |
|  | 6 | 59.2 | 96.7 | 99.9 | 18.4 | 40.4 | 61.2 | 14.6 | 14.1 | 16.2 |
|  | 12 | 63.3 | 96.5 | 99.9 | 20.9 | 40.6 | 59.2 | 13.7 | 15.3 | 17.4 |
| 6 Clusters |  |  |  |  |  |  |  |  |  |  |
|  | 2 | 57.5 | 93.4 | 98.3 | 17.7 | 40.4 | 62.5 | 13.9 | 12.6 | 16.3 |
|  | 3 | 62.5 | 97.2 | 99.8 | 23.4 | 41.9 | 66.9 | 10.4 | 13.4 | 16.5 |
|  | 4 | 64.0 | 97.1 | 100.0 | 20.5 | 43.0 | 67.4 | 11.7 | 14.9 | 14.9 |
|  | 6 | 67.3 | 98.2 | 99.8 | 19.5 | 42.7 | 64.3 | 11.7 | 12.1 | 17.0 |
|  | 12 | 66.6 | 98.6 | 100.0 | 22.1 | 40.7 | 63.6 | 12.0 | 12.4 | 14.3 |
| 9 Clusters |  |  |  |  |  |  |  |  |  |  |
|  | 2 | 69.9 | 96.9 | 99.6 | 21.4 | 50.7 | 78.2 | 10.6 | 13.4 | 18.4 |
|  | 3 | 76.4 | 99.2 | 100.0 | 22.4 | 53.3 | 78.8 | 8.9 | 12.7 | 16.9 |
|  | 4 | 77.1 | 99.7 | 100.0 | 21.4 | 54.9 | 78.2 | 10.4 | 13.6 | 17.8 |
|  | 6 | 80.7 | 99.8 | 100.0 | 23.3 | 53.4 | 79.4 | 11.4 | 13.6 | 17.0 |
|  | 12 | 82.2 | 100.0 | 100.0 | 22.5 | 54.0 | 77.5 | 9.7 | 13.7 | 21.0 |
| 12 Clusters |  |  |  |  |  |  |  |  |  |  |
|  | 2 | 77.8 | 99.3 | 100.0 | 23.8 | 59.5 | 86.2 | 9.5 | 12.8 | 18.2 |
|  | 3 | 84.0 | 99.7 | 100.0 | 24.9 | 60.7 | 87.8 | 9.9 | 15.0 | 18.0 |
|  | 4 | 85.7 | 100.0 | 100.0 | 23.3 | 62.9 | 87.0 | 9.1 | 13.2 | 18.3 |
|  | 6 | 90.1 | 100.0 | 100.0 | 27.9 | 62.9 | 87.0 | 9.5 | 13.8 | 19.2 |
|  | 12 | 88.6 | 100.0 | 100.0 | 25.3 | 63.4 | 87.0 | 8.4 | 13.8 | 18.1 |

**2.0 Statistical analysis**

Marginal effects Poisson regression models as well as random intercept and random coefficient generalised linear mixed effects Poisson models were used in order to identify the most appropriate model for assessing the impact of the intervention. The relative effect of each covariate on the outcome of interest was assessed while adjusting for all the covariates in the model. Random effects (generalised linear mixed modelling) methods are adopted because of their suitability in the presence of repeated measurements and clustering effects in cluster randomised designs (Murray et al., 1998). Such models are usually used for large numbers of clusters with a small number of repeated measurement times, but were used here to increase the degrees of freedom and to improve statistical power.

To estimate the effectiveness of the study intervention, concurrent comparisons of the outcome measures between the intervention arms using likelihood based approaches were performed. Estimation of the effectiveness of the intervention and investigation of heterogeneity in cluster level TB and ART treatment initiations rates as well as TB and HIV testing uptake rates were investigated using generalised linear mixed effects Poisson models with adjustment for cluster level covariates (baseline outcome measures, proportion of females, distribution of facilities, repeated measurement times, and interaction terms between intervention status and time) were adjusted for. This adjustment reduces between cluster variation in the study outcome measure thereby increasing study power and precision in the estimated effect sizes (Hayes and Moulton, 2009). The most robust method for adjusting for baseline differences in outcome measures and cluster level covariates is to include them in the regression model (Hayes and Moulton, 2009). Because observations from the same cluster are usually correlated, cluster level random effects to account for the autocorrelation between observations were included. This approach allows for both within and between cluster variability (Breslow and Clayton, 1993).

**The statistical model**

If $y_{ij}$ is an outcome response for cluster $j$ $(j=1,\ldots,6)$ at measurement time point $i$ ($i=1,\ldots, T$), which is conditionally Poisson and distributed with mean $\mu_{ij}$, the general statistical model used to assess effectiveness of the intervention is given by

$$y_{ij}|x_{ij},u_{j},u_{ij}\sim Poisson \left( \mu_{ij} \right)$$

$$\log\left( \mu_{ij} \right)= \log\left( n_{ij} \right)+ \beta_{0}+\beta_{1}{treat}_{ij}+\beta_{1}{Base}_{ij}+\beta_{3}{Time}_{ij}+\beta_{4}{treat}_{ij}*{Time}_{ij}+\beta_{5}C_{ij}+u_{j}+u_{j}{treat}_{ij}+u_{ij} (3)$$

where:

$\mu_{ij}$ is the mean count at time *i* for cluster *j*;

$\beta_{0}$ is the grand mean count;

$\beta_{1}$, $\beta_{2}, \beta_{3}, \beta_{4} and \beta_{5}$ are the regressions for the variables included in the model;

${treat}_{ij}$ is an indicator variable for the intervention status (Early=1 and Delayed=0);

${Base}_{ij}$ are the baseline ART and TB treatment initiation rates or testing rates for HIV and TB at baseline;

${Time}_{ij}$ is the repeated measurement time variable at time i=1,...,T in cluster j=1,...,6;;

${treat}_{ij}*{Time}_{ij}$ i$s$an interaction term;

$C_{ij}$ represents any cluster level covariates considered in the model;

$log(n_{ij})$ is an offset and $u_{j}$ and $u_{ij}$ are the random effects with mean 0 and known variance. To compare the longitudinal trends between two intervention arms in either TB and ART treatment initiation rates or TB and HIV testing uptake rates, an interaction term between intervention status and time was included in the model. Thus, $\beta_{1}$ and $\beta_{4}$ in the model are the coefficients of interest as they represent the intervention effect after adjusting for the other covariates and random effects.

In our analyses, as in other cluster level covariates, intervention status, time and interactions between time and intervention status were treated as fixed effects.

Because there were different cluster-level population sizes, $log(n_{ij})$ is an offset (covariate with regression coefficient set to 1) used to obtain model based cluster-specific estimates taking into account the cluster-level population (Rabe-Hesketh and Skrondal, 2012, page 724). The random cluster-level intercept $u_{j}$ accounts for within and between cluster correlations resulting from the repeated measurements within each cluster and the cluster randomised design respectively (Hayes and Moulton, 2009; Hu et al., 1998). The random intercept $u_{ij}$ models the overdispersion in the count data. All the random effects are assumed to be Normally distributed with mean zero and an unknown variance-covariance matrix. The $C_{ij}$ represents any cluster level covariates considered in the model that are likely to affect the outcome of interest (eg distribution of health facilities, gender distribution). Therefore, $\beta_{5}$ in model (3) is a vector of regression coefficients for the covariates included in the $C_{ij}$, and fixed effects $\beta_{0}$ to $\beta_{4}$ are single estimates.

Instead of using generalised estimating equations in determining marginal models, Poisson generalised linear models with robust variance estimators were used to adjust for clustering because of the robustness issues of using generalised estimating equations with a small number of clusters (Donner, Eliasziw and Klar, 1994; Moore and Tsiatis, 1991; Zou, 2004).

**Monthly and cumulative treatment initiations and diagnostic uptake rates**

In addition to the statistical modelling used in assessing the effectiveness of the intervention in increasing TB and HIV treatment initiations and diagnostic uptake, monthly and cumulative access rates for TB and HIV services between the two arms were also calculated and plotted using cluster level populations as denominators. The graphical presentation of the monthly and cumulative treatment and diagnostic uptake rates over time illustrated the differences in TB and HIV treatment initiations and diagnostic uptake rates between the study arms which were then confirmed by fitting the marginal and random effects statistical models with some covariate adjustments.

**Results**

**Below are the results of all the three models.**

| **Table 2:** **Adjusted incidence rate ratios for intervention effects using the marginal effects, random intercept, and random coefficients models for measuring TB treatment initiation rates in the first 12 months of the intervention** | | | | | | | | | |
| --- | --- | --- | --- | --- | --- | --- | --- | --- | --- |
|  | Marginal effects Poisson | | | Random intercept Poisson | | | Random coefficient Poisson | | |
| Fixed part | IRR^1^ | 95% CI^2^ | P^3^ | IRR^1^ | 95% CI^2^ | P^3^ | IRR^1^ | 95% CI^2^ | P^3^ |
| Treat^4^ | 1.204 | 0.957, 1.515 | 0.112 | 1.219 | 0.835, 1.779 | 0.305 | 1.239 | 0.881,1.744 | 0.218 |
| Base^5^ | 1.025 | 1.003, 1.048 | 0.025 | 1.027 | 1.001, 1.053 | 0.040 | 1.025 | 1.000,1.050 | 0.046 |
| Occasion^6^ | 1.004 | 0.978, 1.031 | 0.748 | 1.004 | 0.983, 1.026 | 0.694 | 1.004 | 0.983,1.026 | 0.694 |
| Gender | 1.255 | 0.306, 5.140 | 0.752 | 1.181 | 0.084, 16.50 | 0.902 | 0.912 | 0.107,7.743 | 0.932 |
| Treat*Month^7^ | 0.981 | 0.944, 1.012 | 0.335 | 0.981 | 0.950, 1.014 | 0.253 | 0.981 | 0.950,1.014 | 0.253 |
| H. facility^8^ | 1.247 | 1.096, 1.419 | 0.001 | 1.244 | 1.053, 1.470 | 0.010 | 1.248 | 1.067,1.458 | 0.005 |
| Log likelihood | | -236.975 |  |  | -233.781 |  |  | -232.561 |  |
| AIC | | 483.952 |  |  | 481.108 |  |  | 483.123 |  |
| BIC | | 495.335 |  |  | 494.768 |  |  | 503.613 |  |
| 1 Incidence Rate Ratio (IRR) = rate in Early intervention/rate in delayed intervention; 2 Confidence interval (CI); 3 p-value; 4 Intervention status (Early/Delayed); 5 Baseline TB treatment initiation rates; 6 Repeated measurement time; 7 interaction between intervention status and measurement time; 8 Distribution of health facilities offering TB treatment initiations. | | | | | | | | | |

| **Table 3: Adjusted incidence rate ratios for intervention effects using the marginal effects, random intercept, and random coefficients models for measuring ART initiation rates in the first 12 months** | | | | | | | | | |
| --- | --- | --- | --- | --- | --- | --- | --- | --- | --- |
|  | Marginal effects Poisson | | | Random intercept Poisson | | | Random coefficient Poisson | | |
| Fixed part | IRR^1^ | 95% CI^2^ | P^3^ | IRR^1^ | 95% CI^2^ | P^3^ | IRR^1^ | 95% CI^2^ | P^3^ |
| Treat^4^ | 1.356 | 1.003, 1.833 | 0.048 | 1.341 | 1.053, 1.706 | 0.017 | 1.372 | 1.078, 1.746 | 0.010 |
| Base^5^ | 1.003 | 0.993, 1.013 | 0.567 | 1.003 | 0.994, 1.011 | 0.545 | 0.998 | 0.989, 1.008 | 0.741 |
| Occasion^6^ | 0.992 | 0.962, 1.024 | 0.619 | 0.992 | 0.976, 1.008 | 0.344 | 0.992 | 0.976, 1.008 | 0.344 |
| Gender | 0.319 | 0.002, 44.68 | 0.650 | 0.328 | 0.002, 59.145 | 0.674 | 0.086 | 0.00, 37.145 | 0.429 |
| Treat*Month^7^ | 0.993 | 0.949, 1.040 | 0.778 | 0.993 | 0.971, 1.016 | 0.572 | 0.993 | 0.971, 1.016 | 0.572 |
| H. facility^8^ | 1.194 | 1.069, 1.333 | 0.002 | 1.200 | 1.030, 1.399 | 0.019 | 1.233 | 1.104, 1.378 | <0.001 |
| Model fit indices | |  |  |  |  |  |  |  |  |
| Log likelihood | | -285.874 |  |  | -285.034 |  |  | -283.855 |  |
| AIC | | 581.748 |  |  | 586.068 |  |  | 587.710 |  |
| BIC | | 593.131 |  |  | 604.281 |  |  | 610.477 |  |
| 1 Incidence Rate Ratio (IRR) = rate in Early intervention/rate in delayed intervention; 2 Confidence interval (CI); 3 p-value; 4 Intervention status (Early/Delayed); 5 Baseline ART initiation rates; 6 Repeated measurement time; 7 interaction between intervention status and measurement time; 8 Distribution of health facilities offering ART treatment initiations. | | | | | | | | | |

| **Table 4: Adjusted incidence rate ratios for intervention effects using the marginal effects, random intercept, and random coefficients models for measuring TB testing uptake rates in the first 12 months** | | | | | | | | | |
| --- | --- | --- | --- | --- | --- | --- | --- | --- | --- |
|  | Marginal effects Poisson | | | Random intercept Poisson | | | Random coefficient Poisson | | |
| Fixed part | IRR^1^ | 95% CI^2^ | P^3^ | IRR^1^ | 95% CI^2^ | P^3^ | IRR^1^ | 95% CI^2^ | P^3^ |
| Treat^4^ | 1.215 | 1.071, 1.379 | 0.003 | 1.215 | 1.001, 1.475 | 0.049 | 1.215 | 1.001, 1.475 | 0.049 |
| Base^5^ | 1.004 | 1.004, 1.004 | <0.001 | 1.004 | 0.998, 1.010 | 0.187 | 1.004 | 0.998, 1.010 | 0.187 |
| Occasion^6^ | 0.940 | 0.906, 0.975 | 0.001 | 0.940 | 0.908, 0.973 | <0.001 | 0.940 | 0.908, 0.973 | <0.001 |
| Gender | 0.028 | 0.028, 0.029 | <0.001 | 0.028 | 0.003, 0.233 | 0.001 | 0.028 | 0.003, 0.233 | 0.001 |
| Treat*Month^7^ | 0.948 | 0.913, 0.986 | 0.013 | 0.948 | 0.902, 0.998 | 0.040 | 0.948 | 0.902, 0.998 | 0.040 |
| Diagnostic sites^8^ | 0.920 | 0.919, 0.921 | <0.001 | 0.920 | 0.848, 0.999 | 0.045 | 0.920 | 0.848, 0.999 | 0.045 |
| Model fit indices | |  |  |  |  |  |  |  |  |
| Log likelihood | | -165.317 |  | -165.63 |  |  | -165.82 |  |  |
| AIC | | 340.635 |  | 347.268 |  |  | 349.642 |  |  |
| BIC | | 348.553 |  | 359.936 |  |  | 363.893 |  |  |
| 1 Incidence Rate Ratio (IRR) = rate in Early intervention/rate in delayed intervention; 2 Confidence interval (CI); 3 p-value; 4 Intervention status (Early/Delayed); 5 Baseline TB testing uptake rates; 6 Repeated measurement time; 7 interaction between intervention status and measurement time; 8 Distribution of TB diagnostic sites. | | | | | | | | | |

**References.**

Arnold BF, Hogan DR, Colford jr JM, and Hubbard AE, 2011. Simulation methods to estimate design power: an overview for applied research [online]. *BMC Medical Research Methodology*, 11:94. Available at Http://www.biomedcentral.com/1471-2288/11/94.

Borm GF, Melis RJF, Teerenstra S, and Peer PG, 2005. Pseudo cluster randomization: a treatment allocation method to minimize contamination and selection bias. *Statist. Med.*, 24, 3535–3547.

Breslow NE, and Clayton DG, 1993. Approximate inference in generalised linear mixed models. *Journal of the American Statistical Association*, 88 (421), 9-25.

Burton A, Altman DG, Royston P, and Holder RL, 2006. The design of simulation studies in medical statistics. *Statist. Med.*, 25, 4279-4292.

Cohen J, 1992. A power primer. [Psychol Bull.](http://www.ncbi.nlm.nih.gov/pubmed/19565683), 112(1), 155-159.

Collins LM, Schafer JL, and Kam C, 2001. A comparison of inclusive and restrictive strategies in modern missing data procedures. *Psychological Methods*, 6(4), 330-351.

Dean CB, Ugarte MD, and Militino AF, 2004. Penalized quasi-likelihood with spatially correlated data. *Computation Statistics and Data Analysis*, , 45, 235-248.

Donner A, Eliasziw M, and Klar N, 1994. A comparison of methods for testing homogeneity of proportions in teratological studies. *Statist. Med*., 13, 1253-1264.

Feldman HA, McKinlay SM, and Niknian N, 1996. Batch sampling to improve power in a community trial: experience from the Pawtucket Heart Health Program. *Evaluation Review,* 20 (3), 244-274.

Gelman A, and Hill J, 2007. *Data analysis using regression and multilevel/hierarchical models-Analytical methods for social research*.1st ed. New York: Cambridge University Press.

Hayes R.J and Moulton L.H, 2009. *Cluster randomised trials*. 1st ed. Florida: Chapman & Hall.

Hu FB, Goldberg J, Hedeker D, Flay BR, Pentz MA, 1998. Comparison of population-averaged and subject specific approaches for analyzing repeated binary outcomes. *American Journal of Epidemiology*  147, 694-703.

Maas CJM, and Hox JJ, 2005. Sufficient sample sizes for multilevel modelling. *Methodology*, 1(3), 86-92.

Moineddin R, Matheson FI, and Glazier RH, 2007. A simulation study of sample size for multilevel logistic regression models. *BMC Medical Research Methodology*, 7:34 doi:10.1186/1471-2288-7-34.

Moore DF and Tsiatis A, 1991. Robust estimation of the variance in moment methods for extra-binomial and extra-Poison variation. *Biometrics*, 47, 383-401.

Murray DM, Hannan PJ, Wolfinger RD, Baker WL, and Dwyer JH, 1998. Analysis of data from group-randomised trials with repeat observations on the same group. *Statistics in Medicine,* 17, 1581-1600.

Murray DM , Varnell SP, and Blitstein JL, 2004. Design and analysis of group randomized trials: a review of recent methodological developments . *Am J.Public Health,* 94 (3), 423 – 432.

Omar RZ, and Thompson SG, 2000. Analysis of a cluster randomized trial with binary outcome data using a multi-level model *.Statist*. *Med*., 19, 2675-2688.

Nakagawa S and Schielzeth H, 2010. Repeatability for gaussian and non-Gaussian data: a practical guide for biologists. Biol. Rev, 85,935-956.

Pals Sl, Wiegand RE, and Murray DM, 2011. Ignoring the group in group-level HIV/AIDS intervention trials: a review of reported design and analytic methods. *AIDS*, 25, 989-996.

Parker DR, Evangelou TE, and Eaton CB, 2005. Intraclass correlation coefficients for cluster randomized trials in primary care: the cholesterol education and research trial. *Contemporary Clinical Trials*, 26, 260–267.

Rabe-Hesketh S, and Skrondal A, 2012. *Multilevel and longitudinal modelling using Stata*. 3rd ed. Texas: Stata Press Publication.

Raudenbush SW, and Liu X, 2000. Statistical power and optimal design for multisite randomized trials. *Psychological Methods,* 5, 199-213.

Simwaka BN, Theobald S, Willets A, Salaniponi FML, Nkhonjera P, Bello G, and Squire SB, 2012. Acceptability and Effectiveness of the Storekeeper-Based TB Referral System for TB Suspects in Sub-Districts of Lilongwe in Malawi. *PLoS ONE,*  7(9), e39746. doi:10.1371/journal.pone.0039746.

Torgerson DJ, 2001. Contamination in trials: is cluster randomisation the answer? *BMJ,* 322, 355–357.

Tutz G, and Kauermann G, 2003. Generalised linear random effects models with varying coefficients. *Computational Statistics and Data Analysis,* 43, 13-28.

Zou G. (2004) A modified Poisson regression approach to prospective studies with binary data. *Am J Epidemiol*, 159, 702–706.
