# Supplementary material for: The effect of engaging unpaid informal providers on case detection and treatment initiation rates for TB and HIV in rural Malawi (Triage Plus): A cluster randomised health system intervention trial
Source: PLoS One. 2017 Sep 6;12(9):e0183312. doi: 10.1371/journal.pone.0183312 (PMC5587112; doi:10.1371/journal.pone.0183312)
Supplement: S3 Appendix — (DOC) [file pone.0183312.s003.doc]

**PROJECT TITLE: TB-HIV COMMUNITY TRIAGE-PLUS**

Research for Equity and Community Health (REACH) Trust

P. O. Box 1597,

Lilongwe

Tel/fax: +265 1 751 247

E-mail: [info@reachtrust.org](mailto:info@reachtrust.org)

**RESEARCH TEAM**

**George F A Bello:** MSc in Epidemiology, (1999) London School of Hygiene and Tropical Medicine

**Brian Faragher:** PhD Statistician

**Bertie Squire:** PhD University of Cambridge

**Lifah Sanudi:** MA Sociology (2004) University of Malawi

# 2. ABSTRACT

Proposal acronym: Triage-Plus for TB-HIV

**Background**

TB and HIV continue to be major determinants of morbidity and mortality in the developing world. Restricted access to quality assured, effective health services for these communicable diseases among poor people is a major obstacle to reaching the Millennium Development Goals. Using a randomized, controlled health system intervention trial this project will evaluate means to address the inequity of essential health service provision for TB and HIV by piloting Triage-Plus (an integrating package incorporating adaptable but linked combinations of disease recognition, health communication, treatment, and referral). The informal healthcare providers that have been identified are community-based such as HIV home-based care teams, village health committees, and primary health care such as health surveillance assistants (HSAs). This is based in the knowledge that they are frequently accessed by the poor and vulnerable. Their interaction with, and added value to, the public health service through implementing Triage-Plus will be formalised and documented. Effectiveness will be assessed through conduct of the trial in rural parts of Lilongwe District in Malawi. A sister trial following the same methodology will be conducted contemporaneously in Sudan, but is not the subject of this application.

**The overall hypothesis:**

Triage-Plus (an integrated package incorporating adaptable but linked combinations of disease recognition, health communication, treatment and referral) for TB and HIV can be implemented by a variety of informal healthcare providers to promote effective health care provision for the poor and the vulnerable.

**Specific Research Objectives:**

1. To design a package containing linked elements of disease recognition, health communication, treatment, and referral (Triage-Plus) for TB and HIV which:
   1. can be adapted for use by a variety of community-based health-care providers;
   2. strengthens existing community health care networks;
   3. promotes synergistic and effective linkages with the formal public health service;
   4. complements formal public health services in tasks that can be done by the community;
   5. provides a point of entry for HIV treatment and prevention services;
   6. builds on different patient perspectives to maintain and strengthen the package.
2. To assess the effectiveness of Triage-Plus delivery by different kinds of informal healthcare providers in TB and ART treatment initiations and diagnostic uptake for the two diseases.
3. To build the capacity of developing country research partners to conduct sustainable, policy relevant, health systems research.

**BACKGROUND**

Tuberculosis (TB) and HIV continue to be major determinants of morbidity and mortality in the developing world. Existing barriers to access to quality assured, effective health services for these communicable diseases among poor people is a major obstacle to improved health and to reaching the Millennium Development Goals (MDGs).

Using a randomised, controlled health system intervention trial this project will address the inequity of essential health service provision for TB and HIV by piloting Triage-Plus. Three different kinds of informal healthcare providers have been selected: informal private (small grocery stores), NGO/ community based organisations (CBOs), (e.g. HIV home-based care teams) and primary health care facilities (e.g. ante-natal clinics) in the knowledge that they are frequently accessed by the poor and vulnerable. Their interaction with, and added value to, the public health service through implementing Triage-Plus will be formalised and documented.

The public health sector is heavily overwhelmed by the enormous demand for quality assured health care services from the effects of TB and HIV amongst many communicable diseases. However, there are human resource shortages that challenge the improvement in health care service provision. Triage-Plus is an integrated package incorporating adaptable, but linked combinations of disease recognition, health communication, treatment, and referral for tuberculosis (TB) and Human Immunodeficiency Virus (HIV). The package is designed in such a way that it can be implemented by a variety of informal healthcare providers in order to promote effective provision of health care for the poor and vulnerable in the community.

**Other relevant national and international research activities**

The current proposal is in line with many international research initiatives, including the pro-poor research programme consortia funded by DFID and is a direct complement to expanding research on ARV formulation, delivery and access, and into the means of effective management of TB and HIV co-infection. The proposed project will improve access to emerging interventions for HIV and TB in affected countries. Finally despite widespread calls for participatory interventions[[1]](#endnote-2),[[2]](#endnote-3) as a means to more cost-effective delivery of health care and increased service utilisation, few rigorous evaluations of such interventions have been conducted[[3]](#endnote-4). This project will provide invaluable gold-standard quantitative evidence about the impact and process of participatory community level health system development and implementation.

The research is in line with African Union Road Map and specifically addresses challenges identified by the African Platform for Action adopted by the 5^th^ Regional conference, which specifically calls for strengthening of referral systems, improved household to hospital continuum of care and empowerment of women (See appendix 1).

The National TB Control Programme of the Ministry of Health has embarked on universal access to TB care. This is an initiative that aims at reaching people of all socio-economic and geographical distribution in TB service delivery. Among other strategies, informal healthcare providers will be involved in case-finding. Improvements in efficiency will lead to gains in affordability at the household level and the Triage plus intervention has been specifically designed to optimise equity and quality of service. Triage plus will develop win-win partnerships with these key and often under recognised informal and formal providers of health care.

**LITERATURE REVIEW AND JUSTIFICATION**

Access to TB and HIV services is a challenge to most people who live in hard-to-reach areas. There is an urgent need to engage and strengthen community-based health-care providers to address the needs of the poor and vulnerable. Those who are most vulnerable to communicable diseases and have the greatest need for health care have difficulties in accessing the formal public health services[[4]](#endnote-5). These difficulties include geography, lack of trust in the quality of public health services, and lack of empowerment of women and adolescent girls (as patients and carers) to mobilise adequate and timely resources to access these services. In addition the un-tapped resources of involving expert patients, receiving treatment, and ex- patients in order to address patient needs have not yet been used optimally to improve and strengthen health-care provision.

Poor and vulnerable people in Africa use a variety of formal and informal community-based health care providers for their health needs, especially in the early stages of their quest for health care[[5]](#endnote-6). These providers are, in general, ill-equipped either to communicate reliable health information messages or to provide effective treatment to those who can be treated safely at this level of the system. Furthermore such providers are seldom able to identify those who will benefit from referral to formal public health services. As a result, poor people attending these providers, often repeatedly, waste time and resources and eventually experience poor health outcomes[[6]](#endnote-7).

At the same time, the already-weak formal public health service is less and less able to cope with the burdens of HIV-AIDS and TB, which continue to strain limited health budgets, laboratories and clinics; worsen overcrowding in hospitals; and so further hinder existing efforts to increase access to treatment. Furthermore, most public health services in Africa are facing critical shortages of human resources due to HIV-AIDS attrition and migration to developed countries. In particular, the enormous burden on health care staff in some areas, particularly due to HIV/AIDS, has resulted in high levels of stress and, in some cases, even increased emigration of skilled staff. Engaging and strengthening diverse community-based providers to work synergistically with and within public health services could strengthen the health system overall by:

1. improving the effectiveness of treatment provision at the first point of access;
2. relieving short term demand on the weakened formal health sector;
3. relieving the long term burden arising from delayed access to effective treatment, especially for the poor and vulnerable[[7]](#endnote-8), [[8]](#endnote-9)

Scrutiny of national TB data sets reveals that poorer areas do not report more cases of TB because of access barriers[[9]](#endnote-10). Consequently the most vulnerable, especially women and the very poor often contact several informal providers before attempting to approach the organised public health services by which time they are likely to have exhausted their household resources (see table 1). Storekeepers, traditional healers, and other informal community health advisors are often the first port of call[[10]](#endnote-11),[[11]](#endnote-12),[[12]](#endnote-13),[[13]](#endnote-14),[[14]](#endnote-15) along the health-seeking pathway. Interventions among and involving such informal healthcare providers are increasingly recognised as a key strategy for health improvement for the poor and vulnerable of the community.[[15]](#endnote-16)

Triage-Plus builds on the current state of the art by recognising that:

1. there is potential for synergy in integrating health interventions which have been designed for single diseases. For example it has proved possible to adapt the malaria training materials used amongst store keepers in Kenya for use in TB training amongst store-keepers in Malawi[[16]](#endnote-17).
2. store-keepers represent only one part of a potential network of informal healthcare providers
3. provision of treatment, especially treatment requiring regular dosing over a period of time (as exemplified by Direct Observation of Treatment [DOT] for TB), creates multiple opportunities for dialogue around key health messages particularly for example on the topic of HIV counselling, testing and treatment.
4. Expert patients (both patients on treatment and ex-patients) are an under-utilised resource in strengthening community-based health provision for diseases requiring long-term clinical management.

**The novel concept of Triage-Plus** aims to improve disease recognition, health communication, treatment, and referral at the first point of access in the community by working with individuals and structures known to be the first port of call along the health-seeking pathway for the most poor and vulnerable. Triage-Plus augments and builds upon separately documented malaria and TB research experiences with one group of community-based informal care providers in two countries. In the first place store-keepers, also known as “corner shops”, “bottle-stores”, or (in Kenya) “dukas” have been trained to recognise and treat uncomplicated malaria in children and refer those with evidence of complications to the formal public health service in Kenya^6,7^. Secondly, In South Africa similar group of providers (storekeepers) has provided successful supervision of TB treatment (DOT)[[17]](#endnote-18),[[18]](#endnote-19).

Triage Plus will develop the store keeper experience to improve health care for adults and adolescents by:

1. In addition to storekeepers, incorporate other key informal healthcare providers (antenatal clinics, primary schools and home-based care organisations)
2. Building the links between patients (and their peers and linked volunteers) and informal healthcare providers.
3. Focussing on integrated provision for HIV and TB;

Triage Plus will therefore be an integrated package which can be tailored for use by diverse informal healthcare providers within the formal and informal health sector. As women and girls face the most barriers to access of quality services, Triage Plus will ensure that all its activities are women friendly without being exclusively designed for women. The package will mainstream symptom assessment and recognition practices to enable informal healthcare providers to routinely carry out one or more of the following according to his or her capacity and circumstances and as part of his or her daily activities:-

1. Recognise selected presentations of TB and HIV in adults and adolescents (e.g. chronic cough, weight loss and fevers).
2. Communicate effectively and sympathetically with patients and members of the public to i) encourage and support health seeking behaviour, ii) provide key health messages about TB and HIV (including counselling for HIV testing) and iii) provide reliable information on the services available in the area for these diseases.
3. Collect appropriate diagnostic specimens (e.g. sputum specimens from chronic cough patients, possibly finger-prick or saliva samples for HIV testing)
4. Recommend and provide appropriate symptom-control treatments (such as anti-pyretics and oral rehydration solutions)
5. Refer to, and receive feedback from, local public health services (e.g. TB patients referred for sputum submission, signposting to voluntary counselling and testing centres for HIV).
6. Store TB and HIV medications distributed by the national programmes and supervise/support TB and or HIV treatment.
7. Be accountable to local patient groups, their peers and associated volunteers.

In this way, Triage-Plus takes the concept of “community mobilisation’ to a new level by empowering different community-based providers to act as more than directors of referral patterns and encourages them to interact, engage with, and augment, formal public health service provision.

**Choosing the key points of entry to the health system that are used by the poor and vulnerable:**

This project aims to work with 3 kinds of informal healthcare providers (appendix 2) as follows:

1. *Primary Care:* Nurse midwives and auxiliary nurses in health centre antenatal clinics
2. *Informal private sector (self treatment):* Grocery stores and informal traders
3. *Home Based care/ Community Organisations:* Home based care, patient- and NGO- groups, including faith-based organisations already providing for the needs of chronically ill, mainly HIV/AIDS patients at community level and primary school teachers, often acting as respected sources of information at community level.

These levels have been chosen because they are known (often primary) access points for the poor and vulnerable (especially women). The effectiveness of the introduction of Triage-Plus and its potential for formalising the involvement of community-based care providers in health care provision will be assessed.

**HYPOTHESIS**

By using a Health Systems approach, Triage Plus will:

1. Improve the accessibility to effective communicable disease health responses at key entry points by involving *Antenatal care, Informal private sector, Home Based care/ Community Organisations, Primary Schools, Patient and NGO groups,* in health promotion, referral, treatment and monitoring for TB and HIV.
2. Improve health care management by strengthened cross-sectoral working and improved interfaces between health providers in the formal and informal sectors, including strengthened community and patient group responses to communicable diseases.

**Innovation-related activities**

In contrast to other health strategies that rely on a top-down approach, Triage Plus builds on the knowledge and experience of community providers and patients and focuses on those agents most relevant to the health of the poor.

The flexibility of our approach allows for adaptation by other key agents (e.g traditional healers or primary schools) if the circumstances are appropriate.

**AIM**

- To improve disease recognition, health communication, treatment, and referral at the first point of access in the community by working with individuals and structures known to be the first port of call along the health-seeking pathway for the most poor and vulnerable.

**OBJECTIVES**

- 1. To design a package containing linked elements of disease recognition, health communication, treatment, and referral (Triage-Plus) for TB and HIV which:

1. can be adapted for use by a variety of community-based health-care providers
2. strengthens existing community health care networks
3. promotes synergistic and effective linkages with the formal public health service
4. relieves formal public health services from tasks that can be done by the community
5. provides a point of entry for HIV treatment and prevention services
6. builds on different patient perspectives to maintain and strengthen the package
   1. To document processes of the implementation of the Triage-Plus package
   2. To assess the effectiveness and cost-effectiveness of Triage-Plus delivery by different kinds of informal healthcare providers in terms of favourable treatment outcomes for TB and TB and HIV integration.
   3. To build the capacity of the community to improve TB and HIV service delivery at primary health care level
   4. To disseminate the findings to policy makers with a view to inform them on what worked well and how to replicate in other areas across the country

**STUDY DESIGN**

**Project type**

This is a health systems research aimed at exploring ways of improving both case-finding and treatment for TB and HIV and AIDS. The primary aim is to develop and test a policy intervention, whereby communities will be encouraged to find ways appropriate to their own particular circumstances to (a) persuade individuals who may have TB and/or HIV to more easily obtain and earlier diagnosis of their condition and (b) to provide them with the support/encouragement needed to start and complete an appropriate treatment regimen.

The project will take a multi-disciplinary approach which builds on the existing strengths and experience of the research partners. The partners are the Liverpool School of Tropical Medicine (LSTM) who will bring technical expertise in disease control, health economics and gender equity. The Norwegian Health and Lung Patients Organisation (LHL) will bring experience in supporting and promoting patient participation and patient organisations in Malawi. Epi-Lab of the Sudan will bring epidemiological and survey expertise in the project implementation process. Through this initiative the partners will share and apply their skills and experience in a range of disciplines including clinical and public health medicine; systematic research synthesis, health systems development; diagnostics, participatory approaches, gender and equity analysis, survey methods, qualitative research and health economics. The project will have two components namely; intervention package and evaluation or research package.

**Intervention package**

The intervention package of the project will encompass several processes aimed at recognising the capacity of the informal healthcare providers, identifying areas of linkage with the district health system and building capacity of both health workers and the informal healthcare providers. Triage-Plus recognises that different informal healthcare providers will not all have the same capacity to deliver all elements of Triage-Plus. The expected pattern of capacities is illustrated in Table 1 but it is clearly recognised that actual capacity will have to be assessed in each setting and may vary according to the setting. A checklist to assess the capacity of the different providers was developed (See appendix 3).

**Table 1:** Matrix for work-packages for informal healthcare providers

| **Element of T+** | **TB** | **HIV** |
| --- | --- | --- |
| **Disease recognition** | 1.Symptom recognition for those presenting to informal healthcare providers  2. identification of chronic coughers in the community | 1. Recognition of HIV syndromes   - Loss of weight - Oral thrush - Shingles - Recurrent diarrhoea |
| **Health communication** | 1.communication within community about TB infection, transmission etc  2. communication with patients regarding stigma  3. health communication re co-infection with HIV | 1.Addressing stigma at community level  2. Safe sex messages  3. Activities with youths  4. Counselling and testing |
| **Diagnostic specimen collection/testing** | 1.community based sputum collection  2. community based sputum transport  3. transport of lab results to patients | 1. Finger-prick HIV testing? 2. Saliva testing? |
| **Treatment supervision and support** | 1. Treatment supervision  2. Group counselling  3. Cotrimoxazole Preventive Treatment  4. Isoniazid Preventive Therapy | 1. psychosocial support pre and post test  2. ARV side-effect support  3. treatment supervision  4. follow-up of ART defaulters |
| **Referral** | 1. identification of chronic coughers  2. Referral for treatment initiation  3. referral for VCT | 1. Referral of syndromic patients for VCT  2. Referral of HIV-positives for treatment initiation  3. Referral of HIV positive cases for Isoniazid Preventive Therapy |

The project will adopt a participatory approach to ensure ownership at both district and community levels. The participatory approach will include mapping of the informal healthcare providers, exploring with them through semi-structured interviews the ways for potential involvement in the project activities as outlined in table 1. The development and pre-testing of IEC materials for the project will be done with the community to ensure accuracy of meaning and cultural acceptability of the messages. At district level, the health workers will be involved in the development of the training materials for and training of the informal healthcare providers. Some IEC experts from the national level will be involved in the development of the training materials for the project.

All key stakeholders for the informal healthcare providers namely; primary health care, informal private sector and community-based organisations will potentially be involved in the project. The primary health care providers include health surveillance assistants (HSAs), nurse midwives and auxiliary nurses in health centre antenatal clinics. The informal private sector comprises grocery stores and informal traders while the community-based organisations include home-based care groups, patient- and NGO- groups such as faith-based organisations already providing for the needs of chronically ill, mainly HIV/AIDS patients at community level and primary school teachers, often acting as respected sources of information at community level.

The capacity and willingness of the different community-based providers within the selected intervention sites will be assessed. The capacity of the providers will be assessed by considering their current activities, the process through which they were formed, affiliation to or networks with other institutions, funding mechanism and demographic information of the groups in terms of composition by sex, education and sex of the group leaders (see appendix 3). The patient’s and patient organisations will be included in decision-making processes about which are the most appropriate informal healthcare providers with which to engage, bearing in mind their needs and the components of the project they would like to undertake among the available elements of disease recognition, health communication, diagnostic specimen collection and testing, treatment and support, and referral for TB and HIV services.

**Mobilisation of community and monitoring of project activities**

The entry point in the communities will be local leaders (village headmen and traditional authorities) and health surveillance assistants (HSAs). These are people who the community recognise as opinion leaders. The opinion leaders will be advised on the need for close consultation amongst themselves when mobilising the community or organising any activity. The structures that these people use to mobilise the people will be used to mobilise the communities. The community members will be involved in the development of the IEC materials. This will be done to ensure that the message is clear and is culturally acceptable by the community. Pre-testing of the messages will be also be done with the community to check the meaning and acceptability from the community’s perspective.

The district health office (DHO) will be playing advisory role during implementation of the project. Staff from the DHO will form part of facilitating team during training of informal healthcare providers. Since the intervention activities will be taking place in the DHOs area of jurisdiction, the officers will be involved at every step of the project development and implementation. As such, the HSAs at every health facility will be supporting REACH Trust staff in monitoring the project activities in the health facility catchment area.

**Project period**

The project is expected to run for five years (56 months; May 2008 – December 2012). The first two years will be for planning and setting up and the actual intervention will start in the third year.

**Project site**

The project will be implemented in rural parts of Lilongwe district. Ideally, the study region would be divided into “clusters”, each consisting of geographically delineated area (defined by a combination of at least 2 traditional authorities). The clusters would then be randomised to either receive the intervention (Early intervention arm) or to act as a control, (Delayed intervention arm) using stratification methods to ensure that the two sets of clusters would be as similar as possible in terms of their poverty profile, population structure, health service coverage, and presence of significant number of at least one of the selected community-based health care providers (store-keepers, ante-natal care clinic and home-based care organisations).

The problem with this methodology is that people in the study clusters do not always attend their “local” health centre, so there would be considerable problems of “pollution” between the two study groups, in that people living in intervention clusters would attend control cluster health centres, and vice versa. While it would be technically relatively simple to identify the location of participants’ homes (and hence do an “intention-to-treat” analysis with individuals assessed according to their actual residence address rather than health centre attended), this would not eliminate the likelihood that some people resident in control clusters would be exposed to the intervention and some people resident in intervention clusters would not be exposed to it. The decision was thus taken to divide the rural parts of Lilongwe district into just six groups with large population sizes of at least 166,000. A random number will be generated in Excel to determine which clusters will receive the intervention early and which will ones will receive the intervention later. Thus, the six study clusters will equally be randomised to the Early intervention arm (to receive the intervention early in the first 12 months) and the Delayed intervention arm (to receive the intervention after 12 months). The 6 clusters will be pair-matched according to population sizes and their proximity to the urban areas.

Because the intervention is to be implemented at community level, allocation of the intervention will not be blinded to the communities and the research team implementing the intervention. However, allocation of TB and HIV cases to the clusters will be blinded.

**Research packages**

The purpose of the research component of the project is to assess feasibility and measure effectiveness of the intervention package. The research will be done based on the pre-determined indicators and processes as outlined in the sections that follow.

**Project indicators**

Effectiveness will be measured by comparing quantitative health impact indicators for TB and HIV over the implementation period of Triage-Plus. The demographic and health survey (DHS) report of 2004 used the following defining characteristics and impact indicators: basic population; age composition and structure; gender, level of education, TB, HIV and poverty prevalence. Other indicators will be the number of cases identified, the proportion of cases put on single treatment and the proportion cases put on dual treatment. The same impact indicators will be measured in this study at baseline and on- going till the end of the project in both clusters (i.e. where Triage-Plus is and is not implemented). In this way the intervention is both historically and concurrently controlled.

There is a strong recognition that Triage-Plus is, however, a health system intervention, not a new drug. Therefore the methodology incorporates adaptations and additions to the clinical trial approach such as health economics and qualitative process evaluation. The former to analyse the cost effectiveness of the intervention to patients, informal healthcare providers and the formal health system and the latter to document the multiple changes, processes and to provide the context for the trial findings. In addition, an overarching strategy for the whole research process will be continuous involvement of the research partners, participation of the communities involved in the research, and engagement of those who need and will use the research results.

**Data collection process**

*Sample size*

Qualitative research

The actual sample size for the qualitative element of the study is unknown at the outset**.** The sample size will be determined when the “point of saturation” is reached (this is the point at which no new information emerges from each group of respondents). However, indicative figures for the qualitative component are 10 – 20 key informants, 20 semi-structured interviews, and 20 in-depth interviews and twenty-four focus group discussions (six for each respondent category).

Quantitative research

The sample size for the quantitative element of this study needs to provide sufficient power to detect clinically important effect sizes for both TB and HIV. The statistical analysis will thus involve multiple comparisons. The exact number of comparisons of primary interest will be kept to a minimum – but to ensure multiple comparison issues are accounted for, all of the sample size / power calculations detailed below are based on an alpha level of 1% rather than the conventional 5% level.

- - - - 1. TB

*Primary outcome measure*: number of TB patients starting treatment - the expected proportion of smear positive cases starting treatment in the control group is estimated to be 55% - an increase in this proportion to 70% in the intervention group will be considered clinically significant.

WHO estimates that Malawi is detecting less than 50% of smear-positive TB cases nation-wide (the international case detection target is 70%). Based on secondary data collected to date:

- the current catchment population of the Lilongwe District rural health centres in the two study clusters is approximately 1.1 million
- the total number of TB cases registered in these rural health centres for which data is available was 936, of which 329 were smear positives and the remaining 607 were smear negative and other forms of TB.

Thus, assuming that actual TB cases are not rising in the community year on year, the expected number of smear positive TB cases registering for treatment in each study group over a one year period would be (329 / 2 =) 165, with at least as many smear negatives and other forms of TB. As this constitutes 55% of all smear positive TB cases, the actual number of smear positive TB cases occurring in each study group will be (165 / 0.55 =) 300. This will provide 87.5% power to detect an increase in the number of smear positive TB cases registering for treatment from 165 (55%) in the control group to 210 (70%) in the intervention group. Given that 41% of the TB cases were smear positive (WHO, 2009), then 512 TB cases (all forms of TB) were expected to start TB treatment at the end of the intervention in the intervention arm.

- - - - 1. HIV

*Primary outcome measure*: number of HIV-infected patients starting ART – the expected proportion of HIV-infected patients starting ART in this region of Malawi is currently estimated to be 15% - an increase in this proportion to 18% (a 20% proportional increase) in the intervention group would be considered clinically significant.

The population of Malawi is approximately 11 million, of which (approximately) 1.1 million (10%) live in the rural areas of Lilongwe. Estimates taken from Malawi’s 5 year ART Scale-Up plan indicate that 85,000 new patients become eligible for ART each year across the country, so 8,500 new patients become eligible for ART in the rural areas of Lilongwe. Data collected by the REACH Trust indicate that the ART programme starts 1,300 people on treatment in the Lilongwe rural health facilities per year, a treatment initiation rate of (1300 / 8500 =) 15%. Thus, the number of new patients becoming eligible for ART in each of the two study groups will be 4,250. This will provide 86.9% power to detect a 20% increase in the proportion of eligible patients starting ART (in absolute terms, from 15% in the control group to 18% in the intervention group).

It is hoped that the intervention will have a much greater impact on the primary outcome measure, increasing the proportion of eligible patients starting ART in the intervention group well towards the national target figure of 50%.

With a decision to use six large clusters with at least 166,000 population to minimise pollution of the intervention, a proper sample size calculation using simulations will be implemented to guide the analysis plan. The simulation studies will be carried out before the analysis of the study.

*Data collection tools*

Data collection tools for primary and secondary outcome measures

Prior and subsequent to the intervention, the chronic cough registers, TB treatment registers, HTC and ARV Treatment Registers from health facilities in the whole of Lilongwe District (rural and urban) will be audited for patient numbers by health centre catchment area. These data will form the basis for the trial analysis. Place of residence data are crucially important in this study, so that the study staff can locate patients to traditional authorities and health centre catchment areas that are identifiable within either the intervention or the control area. The study staff will, therefore, conduct initial training and continuing quality assurance checks on these registers throughout the study.

Data collection tools for health economic evaluation

A questionnaire survey will be carried out on a random sample of 166 rural new smear positive TB patients (83 at each site) who have registered for treatment (or both) and 200 rural patients who have recently (within last 2 months) registered for ART at the beginning of the intervention and at the end of the trial period. A patient who has both started treatment for smear positive TB and who is registered for ART will count for both samples, therefore the total sample size may be less than 366. These surveys will capture HIV and TB patients’ pathways to care and collect data on individuals’ household assets and on health-seeking experiences around current symptoms, identifying where and why care was sought (appendix 4). Using a cost-effectiveness check-list data will be collected on staff, consumables, transport and other costs incurred by the health service in this intervention.

Data collection for process evaluation

Throughout the duration of the project, detailed monthly reports will be compiled and all minutes will be recorded. All individuals and organisations involved, their roles and their processes of engagement will be documented. Key informant interviews. Semi-structured interviews and in-depth interviews will also be done.

*Data analysis*

Qualitative data will be transcribed verbatim. The transcribed data will be saved as rich text file (RTF) format which will be imported in MAXQDA. It will be organised according to themes for analysis.

The quantitative data will be collected and stored using pre-prepared Excel, Access and/or EpiInfo software databases. The characteristic of the control and intervention groups will initially be summarised and compared using standard univariate statistical methods (Fisher exact test and Student t-test/Mann-Whitney U-tests for categorical and continuous measures respectively). Effect sizes for the primary and main secondary measures will be summarised using incidence rate ratios with their 95% confidence intervals. Poisson regression methods will be used to investigate in greater detail the factors influencing the two primary outcome measures, and to adjust the effect estimates for confounding factors. The analyses will be conducted using the SPSS and Stata statistical packages at the REACH Trust, with supervision and advice as necessary provided by Dr. Brian Faragher (Senior Lecturer in Medical Statistics, Liverpool School of Tropical Medicine).

**SIGNIFICANCE OF EXPECTED FINDINGS**

# Policy relevance

This research will provide tools to (a) improve accessibility and quality of first-contact services for TB and HIV; (b) strengthen health systems through improved inter-provider interfaces; (c) focus on the most vulnerable groups in the poorest countries in the world; and (d) strengthen community participation and patient group interfaces with different providers. Malawi alongside Sudan have been chosen as linked field sites for conduct of the Triage-Plus research because these are poor countries within which the poor and vulnerable carry a heavy burden of communicable disease morbidity and mortality. Furthermore, because the Norwegian Heart and Lung patient organization (LHL) has pre-existing, strong links with two locally established NGO’s with a strong track record in implementation action research (REACH Trust in Malawi and Epi-Lab in Sudan).

**Poverty reduction through improved access to effective HIV and TB services.**

In the poorer countries in Sub-Saharan Africa (represented for this project by Malawi and Sudan), loss of income has a disproportionately severe impact on the health of the poor. The adverse economic impacts of TB and HIV are profound.

- TB sufferers are unable to work for an average of 3-4 months and lose around 20 per cent of annual household income as a result. Furthermore, the long and expensive treatment for TB can cost from 5 to 21% of annual household income. The macroeconomic implications are substantial, as 75 per cent of TB cases in developing countries occur among the economically active population (many of whom are women), and estimates suggest that the cost of TB-related productivity losses is significant.
- Terminal AIDS disease costs from 8 to 100% of household income. In Malawi HIV/AIDS combines with the food crisis to promote a vicious cycle: increased malnutrition weakens the resistance of people infected with HIV to opportunistic infections, thereby reducing the workforce available for agricultural and other work.
- The burden of these diseases among women is severe. Women play a vital role in wealth creation especially in the developing world and it has been argued that the ill health of women is one of the biggest obstacles to poverty reduction in the present day.

By improving access to effective services, Triage Plus has the potential to decrease wasteful consumption of resources through inappropriate or harmful therapy and decrease loss of earnings through ill health and so reduce household and macroeconomic poverty. This is in line with the Malawi Growth and Development Strategy (MDGS).

**ETHICAL CONSIDERATIONS**

**The project will follow established standards of ethical research involving patients. It will also aim to safeguard the rights of patients and others involved or affected by the research; transparency with appropriate forms of explanation to study participants;** Informed consent will be sought from all individuals participating in the project; **including young – study participants (even where consent will also be needed from guardians) (See appendix 5); maintaining the highest possible standards of research practices; which applies to research design, data collection, storage, analysis, interpretation and reporting guidelines.**

The project will involve the community in collecting sputum from chronic coughers in some instances. This will present as a challenge and risk for people that will be involved in the project. To reduce risk of infection of the participants, training will be organised for the informal healthcare providers and the package will include issues of safety during sputum collection. In addition, HSAs will be trained to support and supervise the informal healthcare providers’ activities.

Since the people involved in the project will be members of the community, the issue of confidentiality will be crucial. Training for community providers engaged by the project will include stipulations on confidentiality and regular checks that all providers continue to keep the status of the patients they identify and support confidential throughout the trial.

The issue of conducting a test and/or taking results of a test to patients may in some instances raise psychological harm to the patients being tested or getting the results. To decrease the psychological harm caused to the patients, the project will follow the NTP and NAC laid down guidelines when undertaking its project activities. In addition, the informal healthcare providers will be trained in counselling especially amongst those who will be involved in testing.

As an action research, there will be need to collect information of good quality so that the findings give comprehensive understanding of the project activities and findings. To ensure good quality information, the messages for health communication will be pilot tested and feedback will be incorporated in the manual and other training materials that will be developed. There will also be periodic follow-up interviews with members from informal healthcare providers, patients and members of the general community to check quality of information that is being disseminated in the project area.

*Potential benefits*

The community is expected to benefit from the intervention through development of their skills in delivery of services to their clients. There will also be attainment of new skill in areas of referral of TB suspects and HIV cases. New skills will also be gained in areas of disease recognition and care and support of TB and HIV infected people and well as AIDS patients. Members of the community that will be involved in the project will benefit by getting recognition from the community that they will be serving. Above everything else, the community will benefit from being linked up with the formal health service providers. The general community will benefit through awareness of advantages of accessing formal health systems medical services.

The project stands to benefit the patients that would be diagnosed during the project in the intervention area. Among others there will be increased access to good quality information and services. There will also be reduced costs to accessing TB and HIV related services as these services will be brought to the communities. There will also be increased support network within the community as various informal healthcare providers will be brought together to implement the project.

The benefit of the project will also extend to the formal health services providers. The health workers will relieved of work burden as some of the duties of HSAs such follow up of patients and contact tracing will be done by the volunteers from the different informal healthcare providers. The model will provide options for reaching targets for treatment ie TB cure rate, number of people starting on ART and treatment adherence for both TB and ART. Since both TB and HIV programmes are striving to integrate their activities at community level, the model will provide an opportunity of integrating the TB and HIV programmes at community level.

**DISSEMINATION OF RESEARCH FINDINGS**

Research for Equity and Community Health (REACH), the institutional host of the project, links its research to policy and practice through developing close working relationships with policy makers and practitioners and communicating findings using a range of methods to appropriate audiences. The organisation has strong links with community level networks, national as well as international. Nationally, there are links with the National TB control program (NTP), Malaria Control Programme and the HIV Unit of the Ministry of Health. REACH also organises dissemination workshops which pull participants with different expertise but relevant to the project within Malawian. REACH utilises periodic research meetings organised by other research institutions to disseminate findings.

In addition, REACH trust also has links with wider Equi-TB Knowledge program partnership, which has international academic and policy level links. Presentations will be made at international conferences links such as TB Union Conference. Furthermore, to local and international presentations, papers for publication will be submitted to peer-reviewed journals for international audience.

**ORGANISATIONAL FACILITIES**

Research for Equity and Community Health (REACH) Trust is an independent research trust in Malawi which has grown out of a long established collaboration between the Malawi National TB Control Programme, the Department of Sociology, University of Malawi and the Liverpool School for Tropical Medicine.

REACH trust conducts multidisciplinary research to promote equity in health care. It is run by the Director and has a staff compliment of 30 which includes; research managers, senior Researchers, Researchers, Research Assistants and Administration and Finance Staff. The research team includes experts in Sociology, Clinical Sciences and Health Economics.

The trust is well equipped with facilities for research work. Every officer has a computer; either laptop or desk top and are also connected to internet to facilitate literature search and relevant information from websites. It has a saver which is also used for backups

The trust uses different updated software such as Epi-Info, statistical package for social scientist (SPSS), STATA which are mainly used for quantitative data analysis. We also use MAXQDA for qualitative analysis.

**Collaborative agreements:**

The research will be carried out as a true collaborative partnership between research institutions from the south and north. There will be particular emphasis on south-south linkage between REACH of Malawi and Epi-Lab of Sudan.

The project will take a multi-disciplinary approach which builds on the existing strengths and experience of the research partners. The partners in brief are;

- Liverpool School for Tropical Medicine (LSTM) which is the lead organisation and brings technical expertise in disease control, health economics and gender equity.
- Norwegian Heart and Lung Patient Organisation (LHL) brings experience in supporting and promoting patient participation and patient organisations in developing countries, and has supported activities at both REACH and Epi-Lab for many years.
- REACH Trust, Malawi, brings field experience with piloting elements of Triage-Plus and strengths in qualitative social research methods.
- Epidemiological Laboratory (Epi-Lab), Sudan brings epidemiological and survey expertise.

Thus the research organisations will share and apply their skills and experience in a range of disciplines including clinical and public health medicine; systematic research synthesis, health systems development; diagnostics, participatory approaches, gender and equity analysis, survey methods, qualitative research and health economics.

The participatory nature of the involvement of partners at each stage of the action research cycle will lead to improved research capacity among developing country research partners.

**Appendix 1: Health Economy of symptomatic illness modified from[[19]](#endnote-20)**

| **Health related function** | **Unorganised health care economy** | | **Organised health care economy** |
| --- | --- | --- | --- |
|  | Non marketised | Marketised |  |
| *Skilled consultations and treatment* | - Use of health related knowledge by household members and respected community members such as primary school teachers - Some specialised services such as traditional midwifery provided outside market | - Traditional healers - Unlicensed and/ or unregulated health workers - Covert private practice by public health staff | - Public Health Preventative services - Licensed health workers and facilities including ANC providers - Licensed/ regulated NGOs |
| *Medical related goods* | - Household/ community production of traditional medicines | - Sellers of traditional and western drugs | - Government pharmacies - Licensed pharmacies |
| *Physical support of acutely ill, chronically ill and disabled* | - Household care of sick and disabled - Community support for AIDS patients, people with disabilities | - Domestic servants - Unlicensed nursing homes | - Government hospitals - Licensed or regulated hospitals and nursing homes |

Access points covered by this project:

**Appendix 2: Informal healthcare providers and their Indicative capacity**

|  |  | | | |
| --- | --- | --- | --- | --- |
| **Elements of Triage-Plus** | Home-Based Care Organisations | Primary Schools | Grocery Stores | Community ANC Clinics |
| 1. **Disease Recognition** | +++ | + | ++ | +++ |
| 1. **Health communication** | ++ | +++ | +++ | + |
| 1. **Diagnostic specimen collection / testing** | ++ | + | + | +++ |
| 1. **Treatment supervision & support** | +++ | + | ++ | +++ |
| 1. **Referral** | ++ | + | ++ | ++ |

**Appendix 3:** Topic guide for assessing capacity of close to community providers

1. *Formation process – whether or not the CTC was formed on initiative from*
   1. Local leaders
   2. NGOs
   3. Government agencies
   4. Religious institutions
   5. Self formation
2. *Affiliation or networks with*
   1. National associations
   2. Religious institutions
   3. NGOs
   4. Government agencies
   5. Health centre
   6. District assembly
   7. Local structures
3. *Funding mechanisms*
   1. District Assembly
   2. International NGOs
   3. Religious organisations
   4. Income generating activities
   5. Contributions
      1. Monetary
      2. In-kind
4. *Demographic information*
   1. Group composition by sex/age
   2. Sex of group leader
   3. Educational qualification of leader

**Appendix 4: SURVEY QUESTIONNAIRE**

| 101 | TB Registration number |  | ___________ | TBREG |
| --- | --- | --- | --- | --- |
| 102 | Type of TB | *Exclude EPTB, relapses and transfer-ins* | 1. New sm+ PTB | TBTYPE |
|  |  |  | 2. New sm- PTB |  |
|  |  |  | 99. Not Known |  |
| 103 | Type of DOT |  | 1. Ambulatory | DOTTYPE |
|  |  |  | 2. Guardian-based |  |
|  |  |  | 3. In-patient |  |
|  |  |  | 4. Community volunteer |  |
|  |  |  | 99. Not known |  |
| 104 | ART Registration number |  |  | ARTREG |
| 105 | Sex |  | 1. Female | SEX |
|  |  |  | 2. Male |  |
| 106 | Age in years? |  | _ _ | AGE |
| 107 | Current marital status? |  | 1. Single never married | MARITAL |
|  |  |  | 2. Married |  |
|  |  |  | 3. Divorced/separated |  |
|  |  |  | 4. Widowed |  |
|  |  |  | 99. Not Known |  |
| 108 | Normal area of Residence. | *Refer to map, exclude if not normally resident in Lilongwe* | _ _ | RESIDE |
|  |  |  |  |  |
| 109 | Total number of household members? |  | _ _ | HOUSETOT |
| 110 | How many of these are children under 12? |  |  | CHILDTOT |
| 111 | Total number of dependants in household. |  | _ _ | DEPEND |

| 201 | When did you first have symptoms? | *Enter month in figures* | | Month _ _ Year _ _ | WHENSICK |  |
| --- | --- | --- | --- | --- | --- | --- |
| 202 | At the very beginning of your sickness, what symptoms did you have for your illness? |  | | 1. Persistent coughing | SYMPSIGN |  |
|  |  |  |  | 2. Diarrhoea |  |  |
|  |  |  |  | 3. Fever/malungo |  |  |
|  |  |  |  | 4. Night sweats |  |  |
|  |  |  |  | 5. Coughing/spitting blood |  |  |
|  |  |  |  | 6. Breathlessness |  |  |
|  |  |  |  | 7. Chest pains |  |  |
|  |  |  |  | 8. Loss of body weight/kuonda |  |  |
|  |  |  |  | 9. Weakness |  |  |
|  |  |  |  | 10. Pricking in the chest/zibayo |  |  |
|  |  |  |  | 11. Other Specify ------------------------ |  |  |
|  |  |  |  |  |  |  |
| 203 | What did you think you were suffering from? |  | | 1.Cough | SUFFER |  |
|  |  |  |  | 2.TB |  |  |
|  |  |  |  | 3.Chest pains |  |  |
|  |  |  |  | 4.Malaria/malungo |  |  |
|  |  |  |  | 5.Spirit caused illness-vimbuza |  |  |
|  |  |  |  | 6.Bewitchment-kulodza |  |  |
|  |  |  |  | 7.Breaking taboos (kaliondeonde/ tsempho/mdulo) |  |  |
|  |  |  |  | 8.Asthma/breathlessness (befu) |  |  |
|  |  |  |  | 9. Other sp:_____________________________ |  |  |
|  |  |  |  | 99. Not known |  |  |
| 204 | Which major sign prompted you to seek care? | *Circle one* | | 1.Persistent coughing | PROMPT |  |
|  |  |  |  | 2.Persistent coughing with sputum |  |  |
|  |  |  |  | 3.Fever/malungo |  |  |
|  |  |  |  | 4.Night sweats |  |  |
|  |  |  |  | 5. Coughing/spitting blood |  |  |
|  |  |  |  | 6. Breathlessness |  |  |
|  |  |  |  | 7.Chest pains |  |  |
|  |  |  |  | 8. Loss of body weight/kuonda |  |  |
|  |  |  |  | 9. Weakness |  |  |
|  |  |  |  | 10. Pricking in the chest/zibayo |  |  |
|  |  |  |  | 11. Other body pains |  |  |
|  |  |  |  | 12. Other specify__________________ |  |  |
|  |  |  |  | 99. Not known |  |  |
| 205 | What prompted you to get an HIV test | *Circle one* | 1.Persistent coughing | |  | |
|  |  |  | 2.Persistent coughing with sputum | |  |  |
|  |  |  | 3.Fever/malungo | |  |  |
|  |  |  | 4.Night sweats | |  |  |
|  |  |  | 5. Coughing/spitting blood | |  |  |
|  |  |  | 6. Breathlessness | |  |  |
|  |  |  | 7.Chest pains | |  |  |
|  |  |  | 8. Loss of body weight/kuonda | |  |  |
|  |  |  | 9. Weakness | |  |  |
|  |  |  | 10. Pricking in the chest/zibayo | |  |  |
|  |  |  | 11. Other body pains | |  |  |
|  |  |  | 12. Other specify__________________ | |  |  |
|  |  |  | 99. Not known | |  |  |

|  |  |  |  |  | **1st** | **2nd** | **3rd** | **4th** | **5th** | **6th** | **7th** | **8th** | **9th** | **10th** |
| --- | --- | --- | --- | --- | --- | --- | --- | --- | --- | --- | --- | --- | --- | --- |
| 301 | Where did you seek care? | *Go through each stage of the pathway in turn* | 1. Self treat | CARE1 |  |  |  |  |  |  |  |  |  |  |
|  |  |  | 2. Grocery-self treat | CARE2 |  |  |  |  |  |  |  |  |  |  |
|  |  |  | 3. Pharmacy | CARE3 |  |  |  |  |  |  |  |  |  |  |
|  |  |  | 4. Govt facility | CARE4 |  |  |  |  |  |  |  |  |  |  |
|  |  |  | 5. Private clinic | CARE5 |  |  |  |  |  |  |  |  |  |  |
|  |  |  | 6. Traditional healer | CARE6 |  |  |  |  |  |  |  |  |  |  |
|  |  |  | 7. Other (specify) | CARE7 |  |  |  |  |  |  |  |  |  |  |
|  |  |  |  |  |  |  |  |  |  |  |  |  |  |  |
|  |  |  | 99. Not known |  |  |  |  |  |  |  |  |  |  |  |
| 302 | Date the provider was visited? |  |  | DATEVISI |  |  |  |  |  |  |  |  |  |  |
| 303 | Why did you go there? |  | 1. Minor ailment | CAREWHY1 |  |  |  |  |  |  |  |  |  |  |
|  |  |  | 2. Cost low or free treatment |  |  |  |  |  |  |  |  |  |  |  |
|  |  |  | 3. Quick or polite service |  |  |  |  |  |  |  |  |  |  |  |
|  |  |  | 4. Treatment effective for ailment |  |  |  |  |  |  |  |  |  |  |  |
|  |  |  | 5. Close to home/short distance |  |  |  |  |  |  |  |  |  |  |  |
|  |  |  | 6. Referral |  |  |  |  |  |  |  |  |  |  |  |
|  |  |  | 7. Recommended (others or media) |  |  |  |  |  |  |  |  |  |  |  |
|  |  |  | 8. No particular reason |  |  |  |  |  |  |  |  |  |  |  |
|  |  |  | 9. Other sp: ________ |  |  |  |  |  |  |  |  |  |  |  |
|  |  |  | 99. Not known |  |  |  |  |  |  |  |  |  |  |  |
| 304 | Why did you go back more than once?  **Ndi chifukwa chiyani munapitanso?** |  | 1.choose to return when not better  2.Instructed to return if not better  3.Instructed to come for more test  4.No attention/treatment on first visit  5.Was referred  6.Other sp:_______  88.Not applicable  99.Not known | **RETURN** |  |  |  |  |  |  |  |  |  |  |
| 305 | Who decided that you should go there? |  | 1. Self 2. Spouse 3. Household head 4. Relatives/friends 5. Employer 6. Storekeeper referred 7. Referral health worker 8. Othersp:_______   99. Not known | DECIDE |  |  |  |  |  |  |  |  |  |  |
| 306 | a) Did you go there with a guardian? | *If No go to 307* | 1.Yes  2.No |  |  |  |  |  |  |  |  |  |  |  |
|  | b**)** If yes specify the sex of the guardian |  |  | GUARD1 |  |  |  |  |  |  |  |  |  |  |
|  |  |  | 1.Male  2.Female |  |  |  |  |  |  |  |  |  |  |  |
| 307 | How much did you spend (on drugs, fees, consultation)? | *Fill in for each visit to the care provider* | MK | FEES1 | MK | MK | MK | MK | MK | MK | MK | MK | MK | MK |
| 308 | How did you get the money? |  | 1.Own money  2. From Spouse  3.From children/parents  4.From friends/relatives  5.Borrowed (without interest)  6.Borrowed (with interest)  7.Begging  8.Sold assets  9.Othersp:__________ | MONEYHOS |  |  |  |  |  |  |  |  |  |  |

|  |  |  |  |  | 1^st^ | 2^nd^ | 3^rd^ | 4^th^ | 5^th^ | 6^th^ | 7^th^ | 8^th^ | 9^th^ | 10th |
| --- | --- | --- | --- | --- | --- | --- | --- | --- | --- | --- | --- | --- | --- | --- |
| 309 | How did you travel? |  | 1. Did not travel | TPTMODE1 |  |  |  |  |  |  |  |  |  |  |
|  |  |  | 2. Walk | TPTMODE2 |  |  |  |  |  |  |  |  |  |  |
|  |  |  | 3. Bus/Minibus | TPTMODE3 |  |  |  |  |  |  |  |  |  |  |
|  |  |  | 4. Ambulance | TPTMODE4 |  |  |  |  |  |  |  |  |  |  |
|  |  |  | 5 Bicycle | TPTMODE5 |  |  |  |  |  |  |  |  |  |  |
|  |  |  | 6 Own car | TPTMODE6 |  |  |  |  |  |  |  |  |  |  |
|  |  |  | 7 Hired car/Taxi/bicycle | TPTMODE7 |  |  |  |  |  |  |  |  |  |  |
|  |  |  | 8. Stretcher | TPTMODE8 |  |  |  |  |  |  |  |  |  |  |
|  |  |  | 9. Wheel barrow/Oxcart | TPTMODE9 |  |  |  |  |  |  |  |  |  |  |
|  |  |  | 10. Other sp: | TPTMODE10 |  |  |  |  |  |  |  |  |  |  |
|  |  |  | 99. Not Known |  |  |  |  |  |  |  |  |  |  |  |
| 310 | How much did you pay for transport (going there and back including for guardian(s))? | *Fill in for each visit to the care provider*  *(if answer is 2 above then skip this question)* | MK | TPTCOST1 | MK | MK | MK | MK | MK | MK | MK | MK | MK | MK |
| 311 | How much time did you take to go and come back (hrs)? | *Fill in for each visit to the care provider* | Hours | TIME1 | HRS | HRS | HRS | HRS | HRS | HRS | HRS | HRS | HRS | HRS |
| 312 | How much did you spend on food and incidentals? | *Fill in for each visit to the care provider* | MK | FOODFEE1 | MK | MK | MK | MK | MK | MK | MK | MK | MK | MK |

|  |  | | |  | |  | |  | 1^st^ | 2^nd^ | 3^rd^ | 4^th^ | 5^th^ | 6^th^ | 7^th^ | 8^th^ | 9^th^ | 10th | |
| --- | --- | --- | --- | --- | --- | --- | --- | --- | --- | --- | --- | --- | --- | --- | --- | --- | --- | --- | --- |
| 313 | How did you get the money to pay for food, incidentals and transport? | | |  | | 1. Own money | | MONEY1 |  |  |  |  |  |  |  |  |  |  | |
|  |  |  |  |  |  | 2. From spouse (gift) | |  |  |  |  |  |  |  |  |  |  |  | |
|  |  |  |  |  |  | 3. From children/parents (gift) | |  |  |  |  |  |  |  |  |  |  |  | |
|  |  |  |  |  |  | 4. From friends/relatives (gift) | |  |  |  |  |  |  |  |  |  |  |  | |
|  |  |  |  |  |  | 5. Borrowed (without interest) | |  |  |  |  |  |  |  |  |  |  |  | |
|  |  |  |  |  |  | 6. Borrowed (with interest) | |  |  |  |  |  |  |  |  |  |  |  | |
|  |  |  |  |  |  | 7. Begging | |  |  |  |  |  |  |  |  |  |  |  | |
|  |  |  |  |  |  | 8. Sold asset | |  |  |  |  |  |  |  |  |  |  |  | |
|  |  |  |  |  |  | 9. Other sp: ____________________ | |  |  |  |  |  |  |  |  |  |  |  | |
|  |  |  |  |  |  | 99. Not known | |  |  |  |  |  |  |  |  |  |  |  | |
| 314 | Total spent at each care provider? | | | *Calculate sum spent seeking care from each care provider (****for interviewer)*** | | MK | | TOTAL1 | MK | MK | MK | MK | MK | MK | MK | MK | MK | MK | |
|  |  |  |  |  |  |  |  |  |  |  |  |  |  |  |  |  |  |  |  |
| 315 | Did you submit sputum? | | | *Fill in for each visit to the care provider* | | 1. Yes | | SPUTUM1 |  |  |  |  |  |  |  |  |  |  | |
|  |  |  |  |  |  | 2. No | |  |  |  |  |  |  |  |  |  |  |  | |
| 316 | When did you submit sputum? | | | *Enter month in figures* | | Date Month Year | | DATSPU |  |  |  |  |  |  |  |  |  |  | |
| 317 | When did you get results? | | | *Enter month in figures* | | Date Month Year | | DATERES |  |  |  |  |  |  |  |  |  |  | |
| 318 | What was the result of the sputum exam? | | | *Fill in for each visit to the care provider* | | 1. Nothing wrong | | RESULT1 |  |  |  |  |  |  |  |  |  |  | |
|  |  |  |  |  |  | 2. Negative | |  |  |  |  |  |  |  |  |  |  |  | |
|  |  |  |  |  |  | 3. Positive | |  |  |  |  |  |  |  |  |  |  |  | |
|  |  |  |  |  |  | 4. No result | |  |  |  |  |  |  |  |  |  |  |  | |
|  |  |  |  |  |  | 99. Don't know | |  |  |  |  |  |  |  |  |  |  |  | |
| 319 | | Did you get tested for HIV |  | | 1. Yes 2. No | |  | | | | | | | | | | | |  |
| 320 | | When did you get tested |  | | Day month year | |  | | | | | | | | | | | |  |
| 321 | | What was the result of the test |  | | 1. Positive 2. Negative 3. Don’t know | |  | | | | | | | | | | | |  |

|  |  |  |  |  |
| --- | --- | --- | --- | --- |
| 401 | How many days have you lost work whilst getting a diagnosis for TB? | *Enter days in figures* | _____ | LOSTWORK |
| 402 | How many days have you lost work whilst seeking an HIV test |  |  | LOSTWORK1 |
| 403 | How has TB and care seeking affected your life? |  | 1.Lost job  2.No money to buy food  3. Stigma  4.No money to school fees for children  5. Reduced income  7. Other sp:_______________ | IMPACT |
| 404 | How has the HIV diagnosis affected your life |  | 1.Lost job  2.No money to buy food  3. Stigma  4.No money to school fees for children  5. Reduced income  7. Other sp:_______________ | IMPACT1 |

406a Do you think the services that are being rendered at the clinic are making any difference to the community?

____________________________________________________________________________________________________________________________________________________________

b What do you think about bringing TB diagnostic services into the community?

c. What do you think about bringing HIV testing services to the community?

____________________________________________________________________________________________________________________________________________________________________________________________________________________

| 501 | What is your relationship to head of your household?  **?** | 1. Myself | HHEAD |
| --- | --- | --- | --- |
|  |  | 2. Spouse |  |
|  |  | 3. Son/daughter |  |
|  |  | 4. Grandchild |  |
|  |  | 5. Father/Mother |  |
|  |  | 6. Other relation |  |
|  |  | 7. Servant |  |
|  |  | 8. Not related |  |
| 502 | Sex of head of household | 1. Male | SEXHEAD |
|  |  | 2. Female |  |
| 503 | How much education does he or she have? | 1. Never | HEADEDUC |
|  |  | 2. Standard 1 to 5 |  |
|  |  | 3. Standard 6 to 8 |  |
|  |  | 4. JCE |  |
|  |  | 5. MSCE |  |
|  |  | 6. Other sp: _______________ |  |

| 504 | What is his or her main activity? | 1. Salaried Professional, technical, managerial | | HEADOCC | | |
| --- | --- | --- | --- | --- | --- | --- |
|  |  | 1. Salaried – other | |  |  |  |
|  |  | 1. Self employed – medium-large business | |  |  |  |
|  |  | 1. Self employed – small business | |  |  |  |
|  |  | 1. Cash crop farming | |  |  |  |
|  |  | 1. Subsistence farming | |  |  |  |
|  |  | 1. Casual labour | |  |  |  |
|  |  | 1. Contract unskilled labour 2. Unemployed 3. Other (specify) ------------------------ | |  |  |  |
|  |  |  |  |  |  |  |
| 505 | What was the highest class completed? | 1. Standard 1 to 4 2. Standard 5 to 8 3. JCE 4. MSCE 5. Above MSCE 6. NONE/NEVER ATTENDED SCHOOL | | MAXCLASS | | |
| 506 | What is the main activity you do to earn a living? | 1. Salaried Professional, technical, managerial | | PRIMLIV | | |
|  |  | 1. Salaried – other | |  |  |  |
|  |  | 1. Self employed – medium-large business | |  |  |  |
|  |  | 1. Self employed – small business | |  |  |  |
|  |  | 1. Casual labour 2. Contract skilled labour 3. Unemployed 4. Other (specify)----------------- | |  |  |  |
| 507 | When you go for care seeking, who, if anybody, replaces your normal activities? | 1. No one  2. Spouse  3. Male child  4.Female child  5. Male friend/relative/parent  6. Female friend/relative/parent | | REPLACE | | |
| 508 | Do you have a motor vehicle? | 1. Yes | | MOTOR | | |
|  |  | 2. No | |  |  |  |
| 509 | What do you use for cooking? | 1. Purchased Firewood 2. Collected firewood 3. Paraffin 4. Charcoal 5. Other specify:_______________ | | FUELSRC | | |
| 510 | Where do you draw water? | | 1. River/stream | | WATERSRC |  |
|  |  |  | 2. Unprotected well | |  |  |
|  |  |  | 3. Borehole/protected well | |  |  |
|  |  |  | 4. Public tap | |  |  |
|  |  |  | 5. Own tap outside house | |  |  |
|  |  |  | 6. Tap inside the house | |  |  |
|  |  |  | 7. Other specify: _________ | |  |  |
| 511 | What is your source of lighting? | | 1. Paraffin | | LITESRC |  |
|  |  |  | 2. Candle | |  |  |
|  |  |  | 3. Electricity | |  |  |

### Thank you very much

**Appendix 5:** Draft Consent form

I am …………………… and I work with REACH Trust as a researcher. The organisation works in collaboration with the National TB control Programme (NTP). The organisation is implementing a project which aims at bringing TB and HIV services closer to the community. REACH Trust work is implemented with approval from the National Health Sciences Research Committee.

In line with the intervention, a research is being conducted to seek views from community members on the project activities. You are being asked to participate in the study. However, you are free not to participate if you wish so. Your participation or refusal to participate will not affect access to services delivered through the project.

Whatever, we will discuss here will be kept confidential and no other people will identify what we will discuss with you.

Would you grant consent for the interview to take place.

**Verbal consent given? YES NO Signed:_________________**

**Date: __ __/ __ __/ __ __**

**CHILOLEZO KUTI TIKUFUNSENI MAFUNSO**

Ine ndine…………………… Ndimagwira nchito ku bungwe la REACH Trust lomwe kale linkadziwika ndi dzina loti TB Knowledge Programme. Bungweli ndi nthambi ya Malawi National TB Control Program (NTP) ku Lilongwe. Bungweli likugwira ntchito kuno ku Area 56 pothandiza kuchepetsa mtunda omwe anthu amayenda pofuna kuyezetsa matenda amene akudwala. Mogwirizana ndi ntchito yomwe yikuchitika bungweli likupanga kafukufuku ndi cholinga chofuna kupeza momwe anthu akuthandizidwira chiyambire cha ntchitoyi, zovuta zimene amakumana nazo komanso chuma chimene amaononga pa nthawi imene akufuna kapena akulandira mankhwala a matenda a TB. Bungweli likugwira ntchito movomerezedwa ndi nthambi yopanga kafukufuku ya Health Science Research Committee.

Inu ndi modzi mwa anthu omwe asankhidwa kuti ticheze nawo mukafukufukuyu. Choti mudziwe ndi chakuti zonse zimene munene tidzazisunga mwachinsinsi. Tikudziwitseninso kuti kuvomera kapena kukana kuchita nawo kufukufukuyu sikukhudzana ndi kalandiridwe kanu ka mankhwala a TB

Ndathokoza kwambiri

**Verbal consent given? YES NO Signed:_________________**

**ENDNOTES**

1. [↑](#endnote-ref-2)
2. [↑](#endnote-ref-3)
3. [↑](#endnote-ref-4)
4. Squire S B, Belaye A K, Kashoti A, Salaniponi F M L, Mundy CJF, Theobald S, Kemp J, “Lost” smear positive pulmonary tuberculosis cases; where are they and why did we lose them? *Int J Tuberc Dis.*  2005; 9:25-31. [↑](#endnote-ref-5)
5. Willetts A, Squire SB, Nhlema-Simwaka B, Theobald S, Salaniponi FML, Kemp JR  Bridging the gap for TB diagnosis among poor women and men: assessing the role of peri-urban storekeepers in Malawi *Int J Tuberc Lung Dis.* 2006 (submitted) [↑](#endnote-ref-6)
6. Addressing Poverty in TB Control. Options for National TB Control Programmes” WHO/HTM/TB/2005.352 [↑](#endnote-ref-7)
7. Bates I, Fenton C, Gruber J, Lalloo D, Medina Lara A, Squire S B, Theobald S, Thomson R, and Tolhurst R “Vulnerability to malaria, tuberculosis, and HIV/AIDS infection and disease. Part 1: determinants operating at individual and household level *Lancet Infect Dis* 2004; 4: 267–77. [↑](#endnote-ref-8)
8. Bates I, Fenton C, Gruber J, Lalloo D, Medina Lara A, Squire S B, Theobald S, Thomson R, and Tolhurst R Vulnerability to malaria, tuberculosis, and HIV/AIDS infection and disease. Part II: determinants operating at environmental and institutional level *Lancet Infect Dis* 2004; 4: 368–75. [↑](#endnote-ref-9)
9. ### Bello G, Nhlema BS, Theobald S, Salaniponi F, Squire SB (2005) Assessing the potential of routine data to promote pro-poor scaling up of TB control: insights from Malawi Presentation at the International Union Against TB and Lung Diseases, November, 2005.

   [↑](#endnote-ref-10)
10. Johansson E, Long NH, Diwan VK, Winkvist A. Gender and tuberculosis control: perspectives on health seeking behaviour among men and women in Vietnam. Health Policy. 2000 May;52(1):33-51. [↑](#endnote-ref-11)
11. Manandhar DS, Osrin D, Shrestha BP, Mesko N, Morrison J, Tumbahangphe KM, et al. Effect of a participatory intervention with women’s groups on birth outcomes in Nepal: cluster-randomised controlled trial. Lancet. 11 Sep 2004;364:970-978. [↑](#endnote-ref-12)
12. Marsh VM, Mutemi WM, Willetts A, Bayah K, Were S, Ross A, Marsh K.

    Improving malaria home treatment by training drug retailers in rural Kenya.

    Trop Med Int Health. 2004 Apr;9(4):451-60. [↑](#endnote-ref-13)
13. Marsh VM, Mutemi WM, Muturi J, Haaland A, Watkins WM, Otieno G, Marsh K.

    Changing home treatment of childhood fevers by training shop keepers in rural Kenya. Trop Med Int Health. 1999 May;4(5):383-9. [↑](#endnote-ref-14)
14. Uplekar M, Juvekar S, Morankar S, Rangan S, Nunn P. Tuberculosis patients and practitioners in private clinics in India. Int J Tuberc Lung Dis. 1998 Apr;2(4):324-9. [↑](#endnote-ref-15)
15. Commission of Macroeconomics and Health. Report of the Commission of Macroeconomics and Health 2001 [http://www.cmhealth.org](http://www.cmhealth.org/) [↑](#endnote-ref-16)
16. Smimwaka B, Strengthening the skills and Capacity of the informal community health system to increase early access to tuberculosis services for poor men and women; The case of the Extending Services to the Community Project in urban Lilongwe, Malawi. PhD Thesis submitted to University of Liverpool. [↑](#endnote-ref-17)
17. Wilkinson D, Davies GR, Connolly C. Directly observed therapy for tuberculosis in rural South Africa, 1991 through 1994. Am J Public Health 1996;86:1094-1097. [↑](#endnote-ref-18)
18. Wilkinson D. High compliance tuberculosis treatment programme is a rural community. Lancet 1994;343:647-648. [↑](#endnote-ref-19)
19. Bloom G and Standing H. *Pluralism and Marketisation in the Health Sector: Meeting Health Needs in Contexts of Social Change in Low and Middle Income Countries.* IDS Working Paper 136 2001

    [↑](#endnote-ref-20)
